# The Lambert Way to Gaussianize Heavy-Tailed Data with the Inverse of Tukey's *h* Transformation as a Special Case

**DOI:** 10.1155/2015/909231

**Published:** 2015-08-25

**Authors:** Georg M. Goerg

**Affiliations:** Department of Statistics, Carnegie Mellon University, Pittsburgh, PA 15213, USA

## Abstract

I present a parametric, bijective transformation to generate heavy tail versions of arbitrary random variables. The tail behavior of this *heavy tail Lambert*  W × *F*_*X*_ random variable depends on a tail parameter *δ* ≥ 0: for *δ* = 0, *Y* ≡ *X*, for *δ* > 0 Y has heavier tails than *X*. For *X* being Gaussian it reduces to Tukey's *h* distribution. The Lambert W function provides an explicit inverse transformation, which can thus remove heavy tails from observed data. It also provides closed-form expressions for the cumulative distribution (cdf) and probability density function (pdf). As a special case, these yield analytic expression for Tukey's *h* pdf and cdf. Parameters can be estimated by maximum likelihood and applications to S&P 500 log-returns demonstrate the usefulness of the presented methodology. The R package LambertW implements most of the introduced methodology and is publicly available on CRAN.

## 1. Introduction

Statistical theory and practice are both tightly linked to Normality. In theory, many methods require Gaussian data or noise: (i) regression often assumes Gaussian errors; (ii) many time series models are based on Gaussian white noise [1–3]. In such cases, a model *ℳ*_*𝒩*_, parameter estimates and their standard errors, and other properties are then studied, all based on the ideal(istic) assumption of Normality.

In practice, however, data/noise often exhibits asymmetry and heavy tails, for example, wind speed data [4], human dynamics [5], or Internet traffic data [6]. Particularly notable examples are financial data [7, 8] and speech signals [9], which almost exclusively exhibit heavy tails. Thus a model *ℳ*_*𝒩*_ developed for the Gaussian case does not necessarily provide accurate inference anymore.

One way to overcome this shortcoming is to replace *ℳ*_*𝒩*_ with a new model *ℳ*_*G*_, where *G* is a heavy tail distribution: (i) regression with Cauchy errors [10]; (ii) forecasting long memory processes with heavy tail innovations [11, 12], or ARMA modeling of electricity loads with hyperbolic noise [13]. See also Adler et al. [14] for a wide range of statistical applications and methodology for heavy-tailed data.

While such fundamental approaches are attractive from a theoretical perspective, they can become unsatisfactory from a practical point of view. Many successful statistical models and techniques assume Normality, their theory is very well understood, and many algorithms are implemented for the simple and often much faster Gaussian case. Thus developing models based on an entirely unrelated distribution *G* is like throwing out the (Gaussian) baby with the bathwater.

It would be very useful to transform a Gaussian random variable *X* to a heavy-tailed random variable *Y* and vice versa and thus rely on knowledge and algorithms for the well-understood Gaussian case, while still capturing heavy tails in the data. Optimally such a transformation should (a) be bijective, (b) include Normality as a special case for hypothesis testing, and (c) be parametric so the optimal transformation can be estimated efficiently.

**Figure 1:**
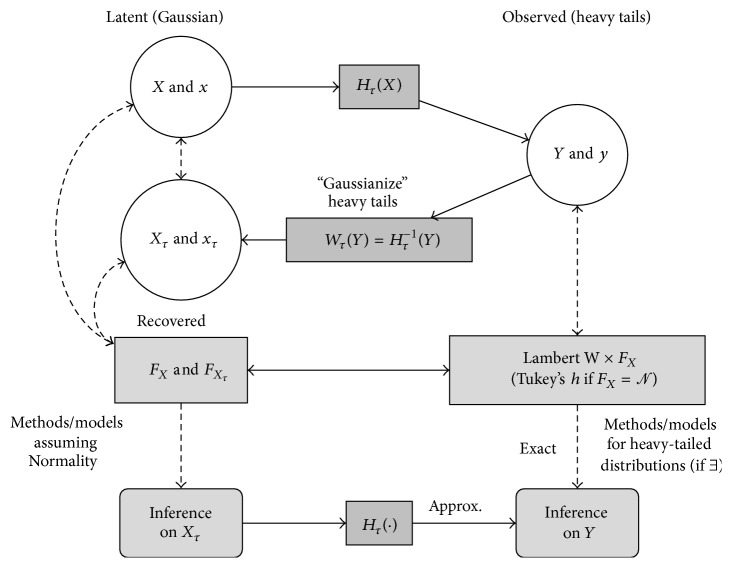
Schematic view of the heavy tail Lambert W ×  *F*_*X*_ framework. Latent input *X* ~ *F*_*X*_: *H*_*τ*_(*X*) from (6) transforms (solid arrows) *X* to *Y*~ Lambert W ×  *F*_*X*_ and generates heavy tails (right) Observed heavy-tailed *Y* and **y**: (1) use *W*_*τ*_(·) to back-transform **y** to latent “Normal” **x**_*τ*_, (2) use model *ℳ*_*𝒩*_ of your choice (regression, time series models, hypothesis testing, etc.) for inference on **x**_*τ*_, and (3) convert results back to the original “heavy-tailed world” of **y** (right).

**Figure 2:**
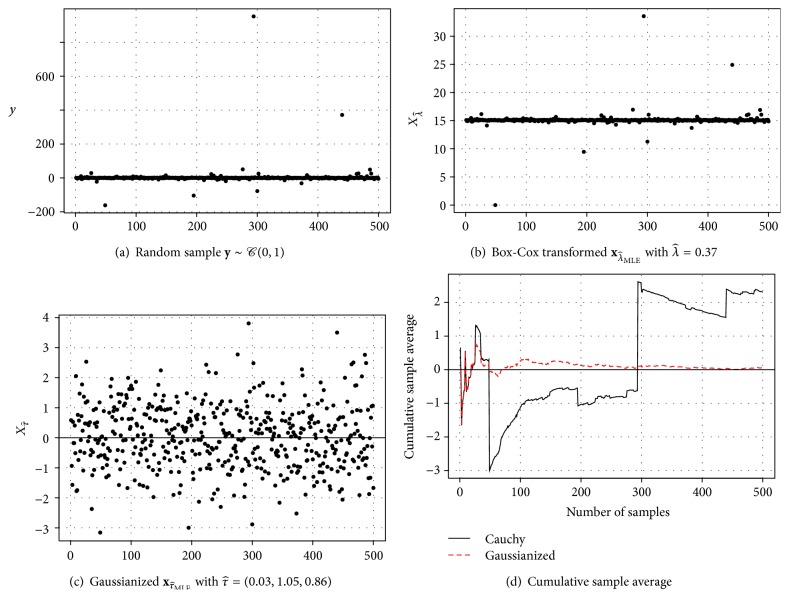
Gaussianizing a standard Cauchy sample. For (d) *τ* ^(*n*)^ was estimated for each fixed *n* = 5,…, 500, before Gaussianizing (*y*_1_,…, *y*_*n*_).

Figure 1 illustrates this pragmatic approach: researchers can make their observations **y** as Gaussian as possible (**x**_*τ*_) before making inference based on their favorite Gaussian model *ℳ*_*𝒩*_. This avoids the development of, or the data analysts waiting for, a whole new theory of *ℳ*_*G*_ and new implementations based on a particular heavy-tailed distribution *G*, while still improving statistical inference from heavy-tailed data **y**. For example, consider **y** = (*y*_1_,…, *y*_500_) from a standard Cauchy distribution *𝒞*(0,1) in Figure 2(a): modeling heavy tails by a transformation makes it even possible to Gaussianize this Cauchy sample (Figure 2(c)). This “nice” data **x**_*τ*_ can then be subsequently analyzed with common techniques. For example, the location can now be estimated using the sample average (Figure 2(d)). For details see Section 6.1.

Liu et al. [15] use a semiparametric approach, where *Y* has a* nonparanormal *distribution if *f*(*Y*) ~ *𝒩*(*μ*, *σ* ^2^) and *f*(·) is an increasing smooth function; they estimate *f*(·) using nonparametric methods. This leads to a greater flexibility in the distribution of *Y*, but it suffers from two drawbacks: (i) nonparametric methods have slower convergence rates and thus need large samples, and (ii) for identifiability of *f*(·), *𝔼f*(*Y*) ≡ *𝔼Y* and Var(*f*(*Y*)) ≡ Var(*Y*) must hold. While (i) is inherent to nonparametric methods, point (ii) requires *Y* to have finite mean and variance, which is often not met for heavy-tailed data. Thus here we use parametric transformations which do not rely on restrictive identifiability conditions and also work well for small sample sizes.

The main contributions of this work are threefold: (a) a metafamily of heavy tail Lambert W × *F*_*X*_ distributions (see also [16]) with Tukey's *h* distribution [17] as a special case, (b) a bijective transformation to “Gaussianize” heavy-tailed data (Section 2), and (c) simple expressions for the cumulative distribution function (cdf) *G*_*Y*_(*y*) and probability density function (pdf) *g*_*Y*_(*y*) (Section 2.4). In particular, analytic expressions for the pdf and cdf for Tukey's *h* (Section 3) are presented here, to the best of the author's knowledge, for the first time in the literature.

Section 4 introduces a method of moments estimator and studies the maximum likelihood estimator (MLE).  Section 5 shows their finite sample properties. As has been shown in many case studies, Tukey's *h* distribution (heavy tail Lambert W × Gaussian) is useful to model data with unimodal, heavy-tailed densities. Applications to S&P 500 log-returns confirm the usefulness of the Lambert W framework (Section 6). Finally, we discuss the new methodology and future work in Section 7. Detailed derivations and proofs are given in the Supplementary Material available online at http://dx.doi.org/10.1155/2015/909231.

Computations, figures, and simulations were done in R [18]. The R package  LambertW implements most of the presented methodology and is publicly available on CRAN.

### 1.1. Multivariate Extensions

While this work focuses on the univariate case, multivariate extensions of the presented methods can be defined component-wise, analogously to the multivariate version of Tukey's *h* distribution [19]. While this may not make the transformed random variables jointly Gaussian, it still provides a good starting point for more well-behaved multivariate estimation.

### 1.2. Box-Cox Transformation

A popular method to deal with skewed, high variance data is the Box-Cox transformation

(1) $$\begin{array}{c} x_{\lambda }=\left\{\begin{array}{cc} \frac{y^{\lambda }-1}{\lambda }, & if\;\;\lambda >0,\\ logy, & if\;\;\lambda =0. \end{array}\right. \end{array}$$

A major limitation of (1) is the nonnegativity constraint on **y**, which prohibits its use in many applications. To avoid this limitation it is common to shift the data, $\tilde{y}=y+\left|min(y)\right|\geq 0$, which restricts *Y* to a half-open interval. If, however, the underlying process can occur on the entire real line, such a shift undermines statistical inference for yet unobserved data (see [20]). Even if out-of-sample prediction is not important for the practitioner, Figure 2(b) shows that the Box-Cox transformation in fact fails to remove heavy tails from the Cauchy sample. (We use $\tilde{y}=y+|min(y)|+1$ and use  boxcox from the  MASS R package; $\hat{\lambda }=0.37$.)

Moreover, the main purpose of the Box-Cox transformation is to stabilize variance [21–23] and remove right tail skewness [24]; a lower empirical kurtosis is merely a by-result of the variance stabilization. In contrast, the Lambert W framework is designed primarily to model heavy-tailed random variables and remove heavy tails from data and has no difficulties with negative values.

## 2. Generating Heavy Tails Using Transformations

Random variables exhibit heavy tails if more mass than for a Gaussian random variable lies at the outer end of the density support. A random variable *Z* has a tail index *a* if its cdf satisfies 1 − *F*_*Z*_(*z*) ~ *L*(*z*)*z* ^−*a*^, where *L*(*z*) is a slowly varying function at infinity, that is, lim_*z*→*∞*_ *L*(*tz*)/*L*(*z*) = 1 for all *t* > 0 [25]. (There are various similar definitions of heavy, fat, or long tails; for this work these differences are not essential.) The heavy tail index *a* is an important characteristic of *Z*; for example, only moments up to order *a* can exist.

### 2.1. Tukey's **h** Distribution

**Figure 3:**
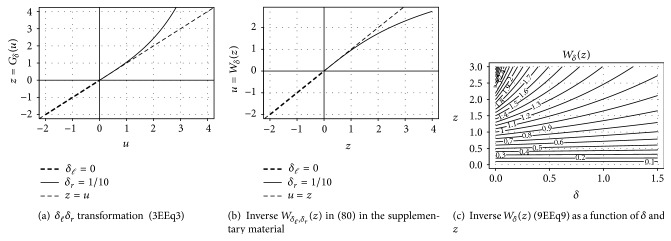
Transformation and inverse transformation for *δ*_*ℓ*_ = 0 and *δ*_*r*_ = 1/10: identity on the left (same tail behavior) and a heavy-tailed transformation in the right tail of input *U*.

A parametric transformation is the basis of Tukey's *h* random variables [17]

(2) $$\begin{array}{c} Z=Uexp\left(\frac{h}{2}U^{2}\right),\;h\geq 0, \end{array}$$

where *U* is standard Normal random variable and *h* is the heavy tail parameter. The random variable *Z* has tail parameter *a* = 1/*h* [17] and reduces to the Gaussian for *h* = 0. Morgenthaler and Tukey [26] extend the *h* distribution to the skewed, heavy-tailed family of *hh* random variables

(3) $$\begin{array}{c} Z=\left\{\begin{array}{cc} Uexp\left(\frac{\delta_{l}}{2}U^{2}\right), & if\;\;U\leq 0,\\ Uexp\left(\frac{\delta_{r}}{2}U^{2}\right), & if\;\;U>0, \end{array}\right. \end{array}$$

where again *U* ~ *𝒩*(0,1). Here *δ*_*ℓ*_ ≥ 0 and *δ*_*r*_ ≥ 0 shape the left and right tail of *Z*, respectively; thus transformation (3) can model skewed and heavy-tailed data; see Figure 3(a). For the sake of brevity let *H*_*δ*_(*u*)∶ = *u*exp((*δ*/2)*u* ^2^).

However, despite their great flexibility, Tukey's *h* and *hh* distributions are not very popular in statistical practice, because expressions for the cdf or pdf have not been available in closed form. Although Morgenthaler and Tukey [26] express the pdf of (2) as (*h* ≡ *δ*)

(4) $$\begin{array}{c} g_{Z}\left(z\right)=\frac{f_{U}\left(H_{\delta }^{-1}\left(z\right)\right)}{H_{\delta }^{\prime }\left(H_{\delta }^{-1}\left(z\right)\right)}, \end{array}$$

they fall short of making *H*_*δ*_^−1^(*z*) explicit. So far the inverse of (2) or (3) has been considered analytically intractable [4, 27]. Thus parameter inference relied on matching empirical and theoretical quantiles [4, 17, 26], or by the method of moments [28]. Only recently Headrick et al. [28] provided numerical approximations to the inverse. However, numerical approximations can be slow and prohibit analytical derivations. Thus a closed form, analytically tractable pdf, which can be computed efficiently, is essential for a widespread use of Tukey's *h* (and variants).

In this work I present this long sought closed-form inverse, which has a large body of literature in mathematics and is readily available in standard statistics software. For ease of notation and concision main results are shown for *δ*_*ℓ*_ = *δ*_*r*_ = *δ*; analogous results for *δ*_*ℓ*_ ≠ *δ*_*r*_ will be stated without details.

### 2.2. Heavy Tail Lambert W Random Variables

Tukey's *h* transformation (2) is strongly related to the approach taken by Goerg [16] to introduce skewness in continuous random variables *X* ~ *F*_*X*_(*x*). In particular, if *Z*~ Tukey's *h*, then *Z* ^2^~ skewed Lambert W ×  *χ*_1_^2^ with skew parameter *γ* = *h*.

Adapting the skew Lambert W ×  *F*_*X*_ input/output idea (see Figure 1), Tukey's *h* random variables can be generalized to* heavy-tailed Lambert *W ×  *F*_*X*_ *random variables*. (Most concepts and methods from the skew Lambert W ×  *F*_*X*_ case transfer one-to-one to the heavy tail Lambert W random variables presented here. Thus for the sake of concision I refer to Goerg [16] for details of the Lambert W framework.)

**Definition 1 .**

Let *U* be a continuous random variable with cdf *F*_*U*_(*u*∣**β**), pdf *f*_*U*_(*u*∣**β**), and parameter vector **β**. Then,

(5) $$\begin{array}{c} Z=Uexp\left(\frac{\delta }{2}U^{2}\right),\;\delta \in R, \end{array}$$

is a* noncentral, nonscaled heavy tail Lambert *W ×  *F*_*U*_ random variable with parameter vector *θ* = (**β**, *δ*), where *δ* is the tail parameter.

Tukey's *h* distribution results for *U* being a standard Gaussian *𝒩*(0,1).

**Definition 2 .**

For a continuous location-scale family random variable *X* ~ *F*_*X*_(*x*∣**β**) define a* location-scale heavy-tailed Lambert *W ×  *F*_*X*_ random variable

(6) $$\begin{array}{c} Y=\left\{Uexp\left(\frac{\delta }{2}U^{2}\right)\right\}\sigma_{X}+\mu_{X},\;\delta \in R, \end{array}$$

with parameter vector *θ* = (**β**, *δ*), where *U* = (*X* − *μ*_*X*_)/*σ*_*X*_ and *μ*_*X*_ and *σ*_*X*_ are mean and standard deviation of *X*, respectively.

**Figure 4:**
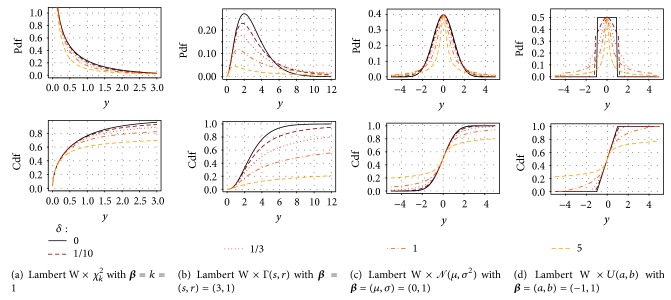
Pdf (left) and cdf (right) of a heavy tail (a) “noncentral, nonscaled,” (b) “scale,” and (c and d) “location-scale” Lambert W ×  *F*_*X*_ random variable *Y* for various degrees of heavy tails (color, dashed lines).

The input is not necessarily Gaussian (Tukey's *h*) but can be any other location-scale continuous random variable, for example, from a uniform distribution, *X* ~ *U*(*a*, *b*) (see Figure 4).

**Definition 3 .**

Let *X* ~ *F*_*X*_(*x*/*s*∣**β**) be a continuous scale-family random variable, with scale parameter *s* and standard deviation *σ*_*X*_; let *U* = *X*/*σ*_*X*_. Then,

(7) $$\begin{array}{c} Y=Xexp\left(\frac{\delta }{2}U^{2}\right),\;\delta \in R, \end{array}$$

is a scaled heavy-tailed Lambert W ×  *F*_*X*_ random variable with parameter *θ* = (**β**, *δ*).

Let *τ*∶ = (*μ*_*X*_(**β**), *σ*_*X*_(**β**), *δ*) define transformation (6). (For noncentral, nonscale input set *τ* = (0,1, *δ*); for scale-family input *τ* = (0, *σ*_*X*_, *δ*).) The shape parameter *δ*( = Tukey′s  *h*) governs the tail behavior of *Y*: for *δ* > 0 values further away from *μ*_*X*_ are increasingly emphasized, leading to a heavy-tailed version of *X*; for *δ* = 0, *Y* ≡ *X*, and for *δ* < 0 values far away from the mean are mapped back again closer to *μ*_*X*_. For *δ* ≥ 0 and *X* ∈ (−*∞*, *∞*), also *Y* ∈ (−*∞*, *∞*). For *δ* ≥ 0 and *X* ∈ [0, *∞*), also *Y* ∈ [0, *∞*).

**Remark 4 (only nonnegative *δ*).**

Although *δ* < 0 gives interesting properties for *Y*, it defines a nonbijective transformation and leads to parameter-dependent support and nonunique input. Thus for the remainder of this work assume *δ* ≥ 0, unless stated otherwise.

### 2.3. Inverse Transformation: “Gaussianize” Heavy-Tailed Data

Transformation (6) is bijective and its inverse can be obtained via the Lambert W function, which is the inverse of *z* = *u*exp(*u*), that is, that function which satisfies *W*(*z*)exp(*W*(*z*)) = *z*. It has been studied extensively in mathematics, physics, and other areas of science [29–31] and is implemented in the GNU Scientific Library (GSL) [32]. Only recently the Lambert W function received attention in the statistics literature [16, 33–35]. It has many useful properties (see Appendix  A in the supplementary material and Corless et al. [29]), in particular, *W*(*z*) is bijective for *z* ≥ 0.

**Lemma 5 .**

The inverse transformation of (6) is

(8) $$\begin{array}{c} W_{\tau }\left(Y\right)∶=W_{\delta }\left(\frac{Y-\mu_{X}}{\sigma_{X}}\right)\sigma_{X}+\mu_{X}=U\sigma_{X}+\mu_{X}=X, \end{array}$$

where

(9) $$\begin{array}{c} W_{\delta }\left(z\right)∶=sgn\left(z\right){\left(\frac{W\left(\delta z^{2}\right)}{\delta }\right)}^{1/2}, \end{array}$$

and sgn(*z*) is the sign of *z*. *W*_*δ*_(*z*) is bijective for all *δ* ≥ 0 and all *z* ∈ *ℝ*.

Lemma 5 gives for the first time an analytic, bijective inverse of Tukey's *h* transformation: *H*_*δ*_^−1^(*y*) of Morgenthaler and Tukey [26] is now analytically available as (8). Bijectivity implies that for any data **y** and parameter *τ*, the exact input **x**_*τ*_ = *W*_*τ*_(**y**) ~ *F*_*X*_(*x*) can be obtained.

In view of the importance and popularity of Normality, we clearly want to back-transform heavy-tailed data to data from a Gaussian rather than yet another heavy-tailed distribution. Tail behavior of random variables is typically compared by their kurtosis *γ*_2_(*X*) = *𝔼*(*X* − *μ*_*X*_)^4^/*σ*_*X*_^4^, which equals 3 if *X* is Gaussian. Hence for the future when we “normalize **y**” we cannot only center and scale but also transform it to **x**_*τ*_ with ${\hat{\gamma }}_{2}\left(x_{\tau }\right)=3$ (see Figure 2(c)). While *γ*_2_(*X*) = 3 does not guarantee that *X* is Gaussian, it is a good baseline for a Gaussian sample. Furthermore, it puts different data not only on the same scale, but also on the same* tail*.

This data-driven view of the Lambert W framework can also be useful for kernel density estimation (KDE), where multivariate data is often prescaled to unit variance, so the same bandwidth can be used in each dimension [36, 37]. Thus “normalizing” the Lambert Way can also improve KDE for heavy-tailed data (see also [38, 39]).

**Remark 6 (generalized transformation).**

Transformation (2) can be generalized to

(10) $$\begin{array}{c} Z=Uexp\left(\frac{\delta }{2}{\left(U^{2}\right)}^{\alpha }\right),\;\alpha >0. \end{array}$$

The inner term *U* ^2^ guarantees bijectivity for all *α* > 0. The inverse is

(11) $$\begin{array}{c} W_{\delta ,\alpha }\left(z\right)∶=sgn\left(z\right){\left(\frac{W\left(\alpha \delta {\left(z^{2}\right)}^{\alpha }\right)}{\alpha \delta }\right)}^{1/2\alpha }. \end{array}$$

For comparison with Tukey's *h* I consider *α* = 1 only. For *α* = 1/2 transformation (10) is closely related to skewed Lambert W ×  *F*_*X*_ distributions.

### 2.4. Distribution and Density

For ease of notation let

(12) $$\begin{array}{c} z=\frac{y-\mu_{X}}{\sigma_{X}},\;\;u=W_{\delta }\left(z\right),\;\;x=W_{\tau }\left(y\right)=u\sigma_{X}+\mu_{X}. \end{array}$$

**Theorem 7 .**

The cdf and pdf of a location-scale heavy tail Lambert W ×  *F*_*X*_ random variable *Y* equal

(13) $$\begin{array}{c} G_{Y}\left(y\;|\;\beta ,\delta \right)=F_{X}\left(W_{\delta }\left(\frac{y-\mu_{x}}{\sigma_{x}}\right)\sigma_{X}+\mu_{X}\;|\;\beta \right), \end{array}$$

(14) gYy ∣ β,δ=fXWδy−μXσXσX+μX ∣ β ·Wδy−μX/σXy−μX/σX1+Wδy−μX/σX2.

Clearly, *G*_*Y*_(*y*∣**β**, *δ* = 0) = *F*_*X*_(*y*∣**β**) and *g*_*Y*_(*y*∣**β**, *δ* = 0) = *f*_*X*_(*y*∣**β**), since lim_*δ*→0_ *W*_*δ*_(*z*) = *z* and lim_*δ*→0_ *W*(*δz* ^2^) = 0 for all *z* ∈ *ℝ*.

For scale family or noncentral, nonscale input set *μ*_*X*_ = 0 or *μ*_*X*_ = 0, *σ*_*X*_ = 1.

The explicit formula (14) allows a fast computation and theoretical analysis of the likelihood, which is essential for statistical inference. Detailed properties of (14) are given in Section 4.1.

Figure 4 shows (13) and (14) for various *δ* ≥ 0 with four different input distributions: for *δ* = *h* = 0 the input equals the output (solid black); for larger *δ* the tails of *G*_*Y*_(*y*∣*θ*) and *g*_*Y*_(*y*∣*θ*) get heavier (dashed colored).

### 2.5. Quantile Function

Quantile fitting has been the standard technique to estimate *μ*_*X*_, *σ*_*X*_, and *δ* of Tukey's *h*. In particular, the medians of *Y* and *X* are equal. Thus for symmetric, location-scale family input the sample median of **y** is a robust estimate of *μ*_*X*_ for any *δ* ≥ 0 (see also Section 5). General quantiles can be computed via [17]

(15) $$\begin{array}{c} y_{\alpha }=u_{\alpha }exp\left(\frac{\delta }{2}u_{\alpha }^{2}\right)\sigma_{X}+\mu_{X}, \end{array}$$

where *u*_*α*_ = *W*_*δ*_(*z*_*α*_) are the *α*-quantiles of *F*_*U*_(*u*).

## 3. Tukey's *h* Distribution: Gaussian Input

For Gaussian input Lambert W ×  *F*_*X*_ equals Tukey's *h*, which has been studied extensively. Dutta and Babbel [40] show that

(16) $$\begin{array}{c} EZ^{n}=\left\{\begin{array}{cc} 0, & if\;n\;is\;odd,n<\frac{1}{\delta },\\ \frac{n!{\left(1-n\delta \right)}^{-\left(n+1\right)/2}}{2^{n/2}\left(n/2\right)!}, & if\;n\;is\;even,n<\frac{1}{\delta },\\ ∄, & if\;n\;is\;odd,n>\frac{1}{\delta },\\ \infty , & if\;n\;is\;even,n>\frac{1}{\delta }, \end{array}\right. \end{array}$$

which, in particular, implies that [28]

(17) $$\begin{array}{c} EZ=EZ^{3}=0,\;if\;\;\delta <1\;and\;\frac{1}{3},\;\;respectively, \end{array}$$

(18) $$\begin{array}{c} EZ^{2}=\frac{1}{{\left(1-2\delta \right)}^{3/2}},\;if\;\;\delta <\frac{1}{2}, \end{array}$$

**Figure 5:**
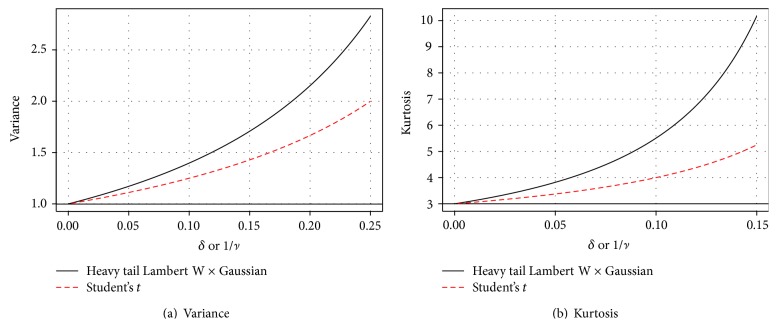
Comparing moments of Lambert W × Gaussian and Student's *t*.

Thus the kurtosis of *Y* equals (see Figure 5)

(19) $$\begin{array}{c} \gamma_{2}\left(\delta \right)=3\frac{{\left(1-2\delta \right)}^{3}}{{\left(1-4\delta \right)}^{5/2}}\;for\;\;\delta <1/4. \end{array}$$

For *δ* = 0, (18) and (19) reduce to the familiar Gaussian values.

Expanding (19) around *δ* = 0 yields

(20) $$\begin{array}{c} \gamma_{2}\left(\delta \right)=3+12\delta +66\delta^{2}+O\left(\delta^{3}\right). \end{array}$$

Dropping *𝒪*(*δ* ^3^) and solving (20) gives a rule of thumb estimate

(21) $$\begin{array}{c} {\hat{\delta }}_{Taylor}=\frac{1}{66}{\left[\sqrt{66\;{\hat{\gamma }}_{2}\left(y\right)-162}-6\right]}_{+}, \end{array}$$

where ${\hat{\gamma }}_{2}(y)$ is the sample kurtosis and [*a*]_+_ = max(*a*, 0); that is, ${\hat{\delta }}_{Taylor}>0$ if ${\hat{\gamma }}_{2}(y)>3$; otherwise, set ${\hat{\delta }}_{Taylor}=0$.

**Corollary 8 .**

The cdf of Tukey's *h* equals

(22) $$\begin{array}{c} G_{Y}\left(y\;|\;\mu_{X},\sigma_{X},\delta \right)=\Phi \left(\frac{W_{\tau }\left(y\right)-\mu_{X}}{\sigma_{X}}\right), \end{array}$$

where Φ(*u*) is the cdf of a standard Normal. The pdf equals (for *δ* > 0)

(23) gYy ∣ μX,σX,δ=12πexp−1+δ2Wδy−μXσX2 ·11+Wδy−μX/σX2.

**Proof**

Take *X* ~ *𝒩*(*μ*_*X*_, *σ*_*X*_^2^) in Theorem 7.

Section 4.1 studies functional properties of (23) in more detail.

### 3.1. Tukey's **h** versus Student's **t**

Student's *t*_*ν*_-distribution with *ν* degrees of freedom is often used to model heavy-tailed data [41, 42], as its tail index equals *ν*. Thus the *n*th moment of a Student's *t* random variable *T* exists if *n* < *ν*. In particular,

(24) $$\begin{array}{c} ET=ET^{3}=0\;if\;\;\nu >1\;\;or\;\;>3,\\ ET^{2}=\frac{\nu }{\nu -2}=\frac{1}{1-\left(2/\nu \right)}\;if\;\;\nu >2, \end{array}$$

and kurtosis

(25) $$\begin{array}{c} \gamma_{2}\left(\nu \right)=3\frac{\nu -2}{\nu -4}=3\frac{1-2\left(1/\nu \right)}{1-4\left(1/\nu \right)}\;if\;\;\nu >4. \end{array}$$

Comparing (24) and (25) with (18) and (19) shows a natural association between 1/*ν* and *δ* and a close similarity between the first four moments of Student's *t* and Tukey's *h* (Figure 5). By continuity and monotonicity, the first four moments of a location-scale *t*-distribution can always be exactly matched by a corresponding location-scale Lambert W × Gaussian. Thus if Student's *t* is used to model heavy tails and not as the true distribution of a test statistic it might be worthwhile to also fit heavy tail Lambert W × Gaussian distributions for an equally valuable “second opinion.” For example, a parallel analysis on S&P 500 log-returns in Section 6.2 leads to divergent inference regarding the existence of fourth moments.

## 4. Parameter Estimation

Due to the lack of a closed form pdf of *Y*, *θ* = (**β**, *δ*) has typically been estimated by matching quantiles or a method of moments estimator [4, 26, 28]. These methods can now be replaced by the, fast and usually efficient, maximum likelihood estimator (MLE). Rayner and MacGillivray [43] introduce a numerical MLE procedure based on quantile functions, but they conclude that “sample sizes significantly larger than 100 should be used to obtain reliable estimates.” Simulations in Section 5 show that the MLE using the closed form Lambert W ×  *F*_*X*_ distribution converges quickly and is accurate even for sample sizes as small as *N* = 10.

### 4.1. Maximum Likelihood Estimation (MLE)

For an i.i.d. sample (*y*_1_,…, *y*_*N*_) = **y** ~ *g*_*Y*_(*y*∣**β**, *δ*) the log-likelihood function equals

(26) $$\begin{array}{c} l\left(\theta ;y\right)=\overset{N}{\underset{i=1}{\sum }}logg_{Y}\left(y_{i}\;|\;\beta ,\delta \right). \end{array}$$

The MLE is that *θ* = (**β**, *δ*) which maximizes (26); that is,

(27) $$\begin{array}{c} {\hat{\theta }}_{MLE}={\left(\hat{\beta },\hat{\delta }\right)}_{MLE}=arg\underset{\beta ,\delta }{max}l\left(\beta ,\delta ;y\right). \end{array}$$

Since *g*_*Y*_(*y*_*i*_∣**β**, *δ*) is a function of *f*_*X*_(*x*_*i*_∣**β**), the MLE depends on the specification of the input density. Equation (26) can be decomposed as

(28) $$\begin{array}{c} l\left(\beta ,\delta ;y\right)=l\left(\beta ;x_{\tau }\right)+R\left(\tau ;y\right), \end{array}$$

where

(29) lβ;xτ=∑i=1NlogfXWδyi−μXσXσX+μX ∣ β=∑i=1NlogfXxτ,i ∣ β

is the log-likelihood of the back-transformed data **x**_*τ*_ = (*x*_*τ*,1_,…, *x*_*τ*,*N*_) (via (8)) and

(30) $$\begin{array}{c} R\left(\tau ;y\right)=\overset{n}{\underset{i=1}{\sum }}logR\left(\mu_{X},\sigma_{X},\delta ;y_{i}\right), \end{array}$$

where

(31) RμX,σX,δ;yi =Wδyi−μX/σXyi−μX/σX1+δWδyi−μX/σX2.

Note that *R*(*μ*_*X*_, *σ*_*X*_, *δ*; *y*_*i*_) only depends on *μ*_*X*_(**β**) and *σ*_*X*_(**β**) (and *δ*), but not necessarily on every coordinate of **β**.

**Figure 6:**
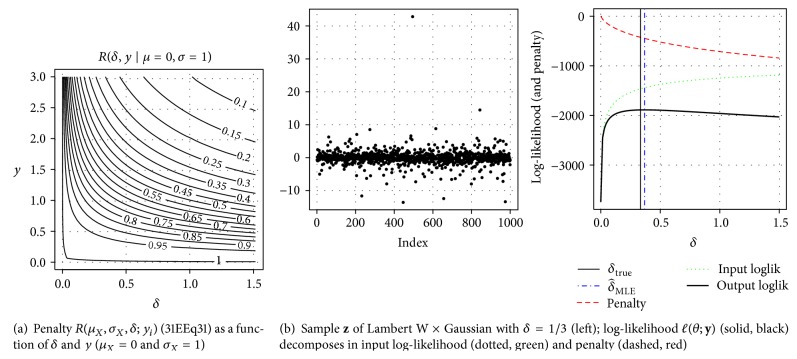
Log-likelihood decomposition for Lambert W ×  *F*_*X*_ distributions.

Decomposition (28) shows the difference between the exact MLE $\left(\hat{\beta },\hat{\delta }\right)$ based on **y** and the approximate MLE ${\hat{\beta }}_{x_{\tau }}$ based on **x**_*τ*_ alone: if we knew *τ* = (*μ*_*X*_, *σ*_*X*_, *δ*) beforehand, then we could back-transform **y** to **x**_*τ*_ and estimate ${\hat{\beta }}_{x_{\tau }}$ from **x**_*τ*_ (maximize (29) with respect to **β**). In practice, however, *τ* must also be estimated and this enters the likelihood via the additive term *ℛ*(*τ*; **y**). A little calculation shows that for any *y*_*i*_ ∈ *ℝ*, log*R*(*μ*_*X*_, *σ*_*X*_, *δ*; *y*_*i*_) ≤ 0 if *δ* ≥ 0, with equality if and only if *δ* = 0. Thus *ℛ*(*τ*; **y**) can be interpreted as a penalty for transforming the data. Maximizing (28) faces a trade-off between transforming the data to follow *f*_*X*_(*x*∣**β**) (and increasing $\ell \left(\beta ;x_{\hat{\tau }}\right)$) and the penalty of a more extreme transformation (and decreasing *ℛ*(*τ*; **y**)); see Figure 6(b).

Figure 6(a) shows a contour plot of *R*(*μ*_*X*_ = 0, *σ*_*X*_ = 1, *δ*; *y*) as a function of *δ* and *y* = *z*: it increases (in absolute value) either if *δ* gets larger (for fixed *y*) or for larger *y* (for fixed *δ*). In both cases, increasing *δ* makes the transformed data *W*_*δ*_(*z*) get closer to 0 = *μ*_*X*_, which in turn increases its input likelihood. For *δ* = 0, the penalty disappears since input equals output; for *y* = 0 there is no penalty since *W*_*δ*_(0) = 0 for all *δ*.

Figure 6(b) shows an i.i.d. sample (*N* = 1000) **z**~ Lambert W × Gaussian with *δ* = 1/3 and the decomposition of the log-likelihood as in (28). Since **β** = (0,1) is known, the likelihood and penalty are only functions of *δ*.  Theorem 9 shows that the convexity of the penalty (decreasing, red) and concavity of the input likelihood (increasing, green) as a function of *δ* holds true in general for any data **z**, and their sum (solid, black) has a unique maximum; here ${\hat{\delta }}_{MLE}=0.37$ (blue, dashed vertical line). See Theorem 9 below for details.

The maximization of (28) can be carried out numerically. Here I show existence and uniqueness of ${\hat{\delta }}_{MLE}$ assuming that *μ*_*X*_ and *σ*_*X*_ are known. Further theoretical results for ${\hat{\theta }}_{MLE}$ remain for future work. Given the “nice” form of *g*_*Y*_(*y*), which is continuous, twice differentiable (under the assumption that *fx*(·) is twice differentiable), the MLE for *θ* = (**β**, *δ*) should have the usual optimality properties, such as being consistent and efficient [44].

#### 4.1.1. Properties of the MLE for the Heavy Tail Parameter **δ**

Without loss of generality let *μ*_*X*_ = 0 and *σ*_*X*_ = 1. In this case

(32)  lδ;z∝−12∑i=1NWδzi2+∑i=1NlogWδziziaaa−∑i=1Nlog1+δWδzi2

(33)  i=−1+δ2∑i=1NWδzi2−∑i=1Nlog1+δWδzi2.

**Theorem 9 (see [14]).**

Let *Z* have a Lambert W × Gaussian distribution, where *μ*_*X*_ = 0 and *σ*_*X*_ = 1 are assumed to be known and fixed. Also consider only *δ* ∈ [0, *∞*). (While for some samples **z** the MLE also exists for *δ* < 0, it cannot be guaranteed for all **z**. If *δ* < 0 (and *z* ≠ 0), then *W*_*δ*_(*z*) is either not unique in *ℝ* (principal and nonprincipal branch) or does not have a real-valued solution in *ℝ* if *δz* ^2^ < *e* ^−1^.)

(a) If
  (34) $$\begin{array}{c} \frac{\sum_{i=1}^{n}z_{i}^{4}}{\sum_{i=1}^{n}z_{i}^{2}}\leq 3, \end{array}$$
 then ${\hat{\delta }}_{MLE}=0$.
 If (34) does not hold, then
(b) ${\hat{\delta }}_{MLE}>0$ exists and is a positive solution to
  (35) $$\begin{array}{c} \overset{N}{\underset{i=1}{\sum }}z_{i}^{2}W^{\prime }\left(\delta z_{i}^{2}\right)\left(\frac{1}{2}W_{\delta }{\left(z_{i}\right)}^{2}-\left(\frac{1}{2}+\frac{1}{1+W\left(\delta z_{i}^{2}\right)}\right)\right)=0. \end{array}$$
(c) There is only one such *δ* satisfying (35); that is, ${\hat{\delta }}_{MLE}$ is unique.

**Proof**

See Appendix  B in the supplementary material.

Condition (34) says that ${\hat{\delta }}_{MLE}>0$ only if the data is heavy-tailed enough. Points (b) and (c) guarantee that there is no ambiguity in the heavy tail estimate. This is an advantage over Student's *t*-distribution, for example, which has numerical problems and local maxima for unknown (and small) *ν* [45, 46]. On the contrary, ${\hat{\delta }}_{MLE}$ is always a global maximum.

Given the heavy tails in **z** one might expect convergence issues for larger *δ*. However, *W*_*δ*_(*Z*) ~ *𝒩*(0,1) for the true *δ* ≥ 0 and close to a standard Gaussian if ${\hat{\delta }}_{MLE}\approx \delta$. Since the log-likelihood and its gradient depend on *δ* and **z** only via *W*_*δ*_(**z**) the performance of the MLE should thus not get worse for large *δ* as long as the initial estimate is close enough to the truth. Simulations in Section 5 support this conjecture, even for ${\hat{\theta }}_{MLE}$.

### 4.2. Iterative Generalized Method of Moments (IGMM)

A disadvantage of the MLE is the mandatory a priori specification of the input distribution. Especially for heavy-tailed data the eye is a bad judge to choose a particular parametric *f*_*X*_(*x*∣**β**). It would be useful to directly estimate *τ* without the intermediate step of estimating *θ* first.

Goerg [16] presented an estimator for *τ* based on iterative generalized methods of moments (IGMM). The idea of IGMM is to find a *τ* such that the back-transformed data **x**_*τ*_ has desired properties, for example, being symmetric or having kurtosis 3. An estimator for *μ*_*X*_, *σ*_*X*_, and *δ* can be constructed entirely analogous to the IGMM estimator for the skewed Lambert W × *F* case. See the Supplementary Material, Appendix  C for details.

IGMM requires less specific knowledge about the input distribution and is usually also faster than the MLE. Once ${\hat{\tau }}_{IGMM}$ has been obtained, the back-transformed $x_{{\hat{\tau }}_{IGMM}}$ can be used to check if *X* has characteristics of a known parametric distribution *F*_*X*_(*x*∣**β**). It must be noted though that testing for a particular distribution *F*_*X*_ is too optimistic as $x_{\hat{\tau }}$ has “nicer” properties regarding *F*_*X*_ than the true **x** would have. However, estimating the transformation requires only three parameters and for a large enough sample, losing three degrees of freedom should not matter in practice.

## 5. Simulations

This section explores finite sample properties of estimators for *θ* = (*μ*_*X*_, *σ*_*X*_, *δ*) and (*μ*_*Y*_, *σ*_*Y*_) under Gaussian input *X* ~ *𝒩*(*μ*_*X*_, *σ*_*X*_^2^). In particular, it compares Gaussian MLE (estimation of *μ*_*Y*_ and *σ*_*Y*_ only), IGMM and Lambert W × Gaussian MLE, and, for a heavy tail competitor, the median. (For IGMM, optimization was restricted to *δ* ∈ [0,10].) All results below are based on *n* = 1,000 replications.

### 5.1. Estimating **δ** Only

Here I show finite sample properties of ${\hat{\delta }}_{MLE}$ for *U* ~ *𝒩*(0,1), where *μ*_*X*_ = 0 and *σ*_*X*_ = 1 are known and fixed.  Theorem 9 shows that ${\hat{\delta }}_{MLE}$ is unique: either at the boundary *δ* = 0 or at the globally optimal solution to (35). Results in Table 1 were obtained by numerical optimization restricted to *δ* ≥ 0 (⇔log*δ* ∈ *ℝ*) using the  nlm function in *R*.

Table 1 suggests that the MLE is asymptotically unbiased for every *δ* and converges quickly (at about *N* = 400) to its asymptotic variance, which is increasing with *δ*. Assuming *μ*_*X*_ and *σ*_*X*_ to be known is unrealistic and thus these finite sample properties are only an indication of the behavior of the joint MLE, ${\hat{\theta }}_{MLE}$. Nevertheless they are very remarkable for extremely heavy-tailed data (*δ* > 1), where classic average-based methods typically break down. One reason lies in the particular form of the likelihood (32) and its gradient (35) (Theorem 9): although both depend on **z**, they only do so through *W*_*δ*_(**z**) = **u** ~ *𝒩*(0,1). Hence as long as ${\hat{\delta }}_{MLE}$ is sufficiently close to the true *δ*, (32) and (35) are functions of almost Gaussian random variables and standard asymptotic results should still apply.

### 5.2. Estimating All Parameters Jointly

Here we consider the realistic scenario where *μ*_*X*_ and *σ*_*X*_ are also unknown. We consider various sample sizes (*N* = 50, 100, and 1000) and different degrees of heavy tails, *δ* ∈ {0,1/3,1, 1.5}, each one representing a particularly interesting situation: (i) Gaussian data (does additional, superfluous, estimation of *δ* affect other estimates?), (ii) fourth moments do not exist, (iii) nonexisting mean, and (iv) extremely heavy-tailed data (can we get useful estimates at all?)

The convergence tolerance for IGMM was set to tol = 1.22 · 10^−4^.  Table 2 summarizes the simulation.

The Gaussian MLE estimates *σ*_*Y*_ directly, while IGMM and the Lambert W × Gaussian MLE estimate *δ* and *σ*_*X*_, which implicitly give ${\hat{\sigma }}_{Y}$ through $\sigma_{Y}(\delta ,\sigma_{X})=\sigma_{X}\cdot (1/\sqrt{{\left(1-2\delta \right)}^{3/2}})$ if *δ* < 1/2 (see (18)). For a fair comparison each subtable also includes a column for ${\hat{\sigma }}_{Y}={\hat{\sigma }}_{X}\cdot (1/\sqrt{{\left(1-2\hat{\delta }\right)}^{3/2}})$. Some of these entries contain “*∞*,” even for *δ* < 1/2; this occurs if at least one $\hat{\delta }\geq 1/2$. For any *δ* < 1, *μ*_*X*_ = *μ*_*Y*_, thus ${\hat{\mu }}_{X}$ and ${\hat{\mu }}_{Y}$ can be compared directly. For *δ* ≥ 1, the mean does not exist; each subtable for these *δ* interprets *μ*_*Y*_ as the median.

*Gaussian Data *(*δ* = 0). This setting checks if imposing the Lambert W framework, even though it is superfluous, causes a quality loss in the estimation of *μ*_*Y*_ = *μ*_*X*_ or *σ*_*Y*_ = *σ*_*X*_. Furthermore, critical values for *H*_0_ : *δ* = 0 (Gaussian) can be obtained. As in the *δ*-only case above, Table 2(a) suggests that estimators are asymptotically unbiased and quickly tend to a large-sample variance. Additional estimation of *δ* does not affect the efficiency of ${\hat{\mu }}_{X}$ compared to estimating solely *μ*. Estimating *σ*_*Y*_ directly by Gaussian MLE does not give better results than the Lambert W × Gaussian MLE.

*No Fourth Moment *(*δ* = 1/3). Here *σ*_*Y*_(*δ*, *σ*_*X*_ = 1) = 2.28, but fourth moments do not exist. This results in an increasing empirical standard deviation of ${\hat{\sigma }}_{y}$ as *N* grows. In contrast, estimates for *σ*_*X*_ are not drifting off. In the presence of these large heavy tails the median is much less variable than Gaussian MLE and IGMM. Yet, Lambert W × Gaussian MLE for *μ*_*X*_ even outperforms the median.

*Nonexisting Mean *(*δ* = 1). Both sample moments diverge, and their standard errors are also growing quickly. The median still provides a very good estimate for the location but is again inferior to both Lambert W estimators, which are closer to the true values and appear to converge to an asymptotic variance at rate $\sqrt{N}$.

*Extreme Heavy Tails *(*δ* = 1.5). As in Section 5.1, IGMM and Lambert W MLE continue to provide excellent estimates even though the data is extremely heavy tailed. Moreover, Lambert W MLE also has the smallest empirical standard deviation overall. In particular, the Lambert W MLE for *μ*_*X*_ has an approximately 15% lower standard deviation than the median.

The last column shows that for some *N* about 1% of the *n* = 1,000 simulations generated invalid likelihood values (NA and *∞*). Here the search for the optimal *δ* led into regions with a numerical overflow in the evaluation of *W*_*δ*_(*z*). For a comparable summary, these few cases were omitted and new simulations added until full *n* = 1,000 finite estimates were found. Since this only happened in 1% of the cases and also such heavy-tailed data is rarely encountered in practice, this numerical issue is not a limitation in statistical practice.

### 5.3. Discussion of the Simulations

IGMM performs well independent of the magnitude of *δ*. As expected the Lambert W MLE for *θ* has the best properties: it can recover the truth for all *δ*, and for *δ* = 0 it performs as well as the classic sample mean and standard deviation. For small *δ* it has the same empirical standard deviation as the Gaussian MLE, but a lower one than the median for large *δ*.

Hence the only advantage of estimating *μ*_*Y*_ and *σ*_*Y*_ by sample moments of **y** is speed; otherwise the Lambert W × Gaussian MLE is at least as good as the Gaussian MLE and clearly outperforms it in presence of heavy tails.

## 6. Applications

Tukey's *h* distribution has already proven useful to model heavy-tailed data, but parametric inference was limited to quantile fitting or methods of moments estimation [4, 27, 28]. Theorem 7 allows us to estimate *θ* by ML.

This section applies the presented methodology on simulated as well as real world data: (i) Section 6.1 demonstrates Gaussianizing on the Cauchy sample from the Introduction, and (ii) Section 6.2 shows that heavy tail Lambert W × Gaussian distributions provide an excellent fit to daily S&P 500 log-return series.

### 6.1. Estimating Location of a Cauchy with the Sample Mean

It is well known that the sample mean $\bar{y}$ is a poor estimate of the location parameter of a Cauchy distribution, since the sampling distribution of $\bar{y}$ is again a Cauchy (see [47] for a recent overview); in particular, its variance does not go to 0 for *N* → *∞*.

Heavy-tailed Lambert W × Gaussian distributions have similar properties to a Cauchy for *δ* ≈ 1. The mean of *X* (*μ*_*X*_) equals the location of *Y* (*c*) due to symmetry around *μ*_*X*_ and *c*, respectively. Thus we can estimate *τ* from the Cauchy sample **y**, transform **y** to $x_{\hat{\tau }}$, get ${\hat{\mu }}_{X}$ from $x_{\hat{\tau }}=W_{\hat{\tau }}(y)$, and thus obtain an estimate for *c* by $\hat{c}={\hat{\mu }}_{X}$.

The random sample **y** ~ *𝒞*(0,1), with pdf *f*(*y*) = 1/*π*(1 + *y* ^2^), in Figure 2(a) has heavy tails with two extreme (positive) observations. A Cauchy ML fit gives $\hat{c}=0.03(0.055)$ and $\hat{s}=0.86(0.053)$ (standard errors in parenthesis). A Lambert W × Gaussian MLE gives ${\hat{\mu }}_{X}=0.03(0.055)$, ${\hat{\sigma }}_{X}=1.05(0.072)$, and $\hat{\delta }=0.86(0.082)$. Thus both fits correctly fail to reject *μ*_*X*_ = *c* = 0.  Table 3(a) shows summary statistics on both samples. Since the Cauchy distribution does not have a well-defined mean, $\bar{y}=2.304(2.101)$ is not meaningful. However, $x_{{\hat{\tau }}_{MLE}}$ is approximately Gaussian and we can use the sample average for inference: ${\bar{x}}_{{\hat{\tau }}_{MLE}}=0.033(0.0472)$ correctly fails to reject a zero location for **y**. The transformed $x_{{\hat{\tau }}_{MLE}}$ features additional Gaussian characteristics (symmetric, no excess kurtosis), and even the null hypothesis of Normality cannot be rejected (*P*-value ≥0.5). Note, however, that Normality for the transformed data is only an empirical approximation; the random variable *W*_*τ*_((*Y* − *μ*_*X*_)/*σ*_*X*_), where *Y* is Cauchy, does* not* have a Normal distribution.

Figure 2(d) shows the cumulative sample average for the original sample and its Gaussianized version. For a fair comparison ${\hat{\tau }}_{MLE}^{\left(n\right)}$ was reestimated cumulatively for each *n* = 5,…, 500 and then used to compute (*x*_1_,…, *x*_*n*_). The transformation works extremely well: data point *y*_49_ is highly influential for $\bar{y}$ but has no relevant effect on ${\bar{x}}_{{\hat{\tau }}_{MLE}^{\left(n\right)}}$. Even for small *n* it is already clear that the location of the underlying Cauchy distribution is approximately zero.

Although it is a simulated example, it demonstrates that removing (strong) heavy tails from data works well and provides “nice” data that can then be analyzed with more refined Gaussian methods.

### 6.2. Heavy Tails in Finance: S&P 500 Case Study

A lot of financial data displays negative skewness and excess kurtosis. Since financial data in general is not i.i.d., it is often modeled with a (skew) Student's *t*-distribution underlying a (generalized) autoregressive conditional heteroskedastic (GARCH) [2, 48] or a stochastic volatility (SV) [49, 50] model. Using the Lambert W approach we can build upon the knowledge and implications of Normality (and avoid deriving properties of a GARCH or SV model with heavy-tailed innovations) and simply “Gaussianize” the returns before fitting more complex, GARCH or SV, models.

**Remark 10 .**

Time series models with Lambert W × Gaussian white noise are far beyond the scope of this work but can be a direction of future research. Here I only consider the unconditional distribution.

**Figure 7:**
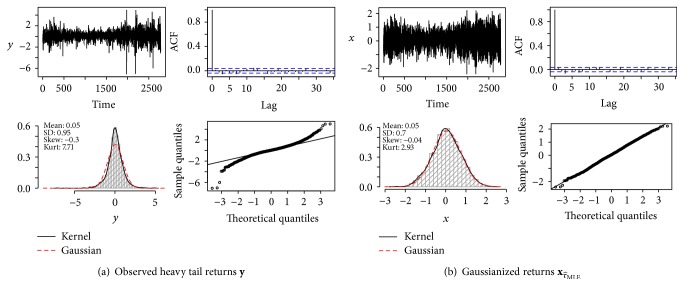
Lambert W Gaussianization of S&P 500 log-returns: $\hat{\tau }=(0.05,0.70,0.17)$. In (a) and (b): data (top left); autocorrelation function (ACF) (top right); histogram, Gaussian fit, and KDE (bottom left); Normal QQ plot (bottom right).

Figure 7(a) shows the S&P 500 log-returns with a total of *N* = 2,780 daily observations (R package  MASS, dataset  SP500).  Table 3(b) confirms the heavy tails (sample kurtosis 7.70) but also indicates negative skewness (−0.296). As the sample skewness ${\hat{\gamma }}_{1}(y)$ is very sensitive to outliers, we fit a distribution which allows skewness and test for symmetry. In case of the double-tail Lambert W × Gaussian this means testing *H*_0_ : *δ*_*ℓ*_ = *δ*_*r*_ = *δ* versus *H*_1_ : *δ*_*ℓ*_ ≠ *δ*_*r*_. Using the likelihood expression in (28), we can use a likelihood ratio test with one degree of freedom (3 versus 4 parameters). The log-likelihood of the double-tail fit (Table 4(a)) equals −3606.0 = −2972.27 + (−633.73) (input log-likelihood + penalty), while the symmetric *δ* fit gives −3606.56 = −2971.47 + (−635.09). Here the symmetric fit gives a transformed sample that is more Gaussian, but it pays a greater penalty for transforming the data. Comparing twice their difference to a *χ*_1_^2^ distribution gives a *P*-value of 0.29. For comparison, a skew-*t* fit [51], with location *c*, scale *s*, shape *α*, and *ν* degrees of freedom, also yields (Function  st.mle in the R package  sn.) a nonsignificant $\hat{\alpha }$ (Table 4(b)). Thus both fits cannot reject symmetry.

Assume we have to make a decision if we should trade a certificate replicating the S&P 500. Since we can either buy or sell, it is not important if the average return is positive or negative, as long as it is significantly different from zero.

#### 6.2.1. Gaussian Fit to Returns

Estimating (*μ*_*Y*_, *σ*_*Y*_) by Gaussian MLE and thus ignoring the heavy tails, ${\hat{\mu }}_{Y}=0$ cannot be rejected on a *α* = 1% level (Table 4(e)). Ignoring heavy tails we would thus decide to not trade a replicating certificate at all.

#### 6.2.2. Heavy Tail Fit to Returns

Both a heavy tail Lambert W × Gaussian (Table 4(c)) and Student *t*-fit (Table 4(d)) reject the zero mean null (*P*-values: 10^−4^ and 3 · 10^−5^, resp.).

Location and scale estimates are almost identical, but tail estimates lead to different conclusions: while for $\hat{\nu }=3.71$ only moments up to order 3 exist, in the Lambert W × Gaussian case moments up to order 5 exist (1/0.172 = 5.81). This is especially noteworthy as many theoretical results in the (financial) time series literature rely on finite fourth moments [52, 53]; consequently many empirical studies test this assumption [7, 54]. Here Student's *t* and a Lambert W × Gaussian fit give different conclusions. Since previous empirical studies often use Student's *t* as a baseline [41], it might be worthwhile to reexamine their findings in light of heavy tail Lambert W × Gaussian distributions.

#### 6.2.3. “Gaussianizing” Financial Returns

The back-transformed $x_{{\hat{\tau }}_{MLE}}$ is indistinguishable from a Gaussian sample (Figure 7(b)) and thus demonstrates that a Lambert W × Gaussian distribution is indeed appropriate for $x_{{\hat{\tau }}_{MLE}}$. Not even one test can reject Normality: *P*-values are 0.18, 0.18, 0.31, and 0.24, respectively (Anderson-Darling, Cramer-von-Mises, Shapiro-Francia, Shapiro-Wilk; see Thode [55]).  Table 3(b) confirms that Lambert W “Gaussianiziation” was successful: ${\hat{\gamma }}_{1}(x_{\hat{\tau }})=-0.039$ and ${\hat{\gamma }}_{2}(x_{\hat{\tau }})=2.93$ are within the typical variation for a Gaussian sample of size *N* = 2780. Thus

(36) Y=Ue0.172/2U20.705+0.055, U=X−0.0550.705,aaaaaaaaaaaaaaaaaaaaaaaaaaaaaaaU~N0,1

is an adequate (unconditional) Lambert W × Gaussian model for the S&P 500 log-returns **y**. For trading, this means that the expected return is significantly larger than zero (${\hat{\mu }}_{X}=0.055>0$) and thus replicating certificates should be bought.

#### 6.2.4. Gaussian MLE for Gaussianized Data

For *δ*_*l*_ = *δ*_*r*_ ≡ *δ* < 1, also *μ*_*X*_ ≡ *μ*_*Y*_. We can therefore replace testing *μ*_*y*_ = 0 versus *μ*_*y*_ ≠ 0 for a non-Gaussian **y**, with the very well-understood hypothesis test *μ*_*x*_ = 0 versus *μ*_*x*_ ≠ 0 for the Gaussian $x_{{\hat{\tau }}_{MLE}}$. In particular, standard errors based on $\hat{\sigma }/\sqrt{N}$ and thus *t* and *P*-values should be closer to the “truth” (Tables 4(c) and 4(d)) than a Gaussian MLE on the non-Gaussian **y** (Table 4(e)). Table 4(f) shows that standard errors for ${\hat{\mu }}_{x}$ are even a bit too small compared to the heavy-tailed versions. Since the “Gaussianizing” transformation was estimated, treating $x_{{\hat{\tau }}_{MLE}}$ as if it was original data is too optimistic regarding its Normality (recall the penalty (30) in the total likelihood (28)).

This example confirms that if a model and its theoretical properties are based on Normality, but the observed data is heavy-tailed, then Gaussianizing the data first gives more reliable inference than applying Gaussian methods to the original, heavy-tailed data (Figure 1). Clearly, a joint estimation of the model parameters based on Lambert W × Gaussian random variables (or any other heavy-tailed distribution) would be optimal. However, theoretical properties and estimation techniques may not be available or well understood. The Lambert Way to Gaussianize data is thus a pragmatic method to improve statistical inference on heavy-tailed data, while preserving the applicability and interpretation of Gaussian models.

## 7. Discussion and Outlook

In this work I use the Lambert W function to model and remove heavy tails from continuous random variables using a data-transformation approach. For Gaussian random variables this not only contributes to existing work on Tukey's *h* distribution but also gives convincing empirical results: unimodal data with heavy tails can be transformed to Gaussian data. Properties of a Gaussian model *ℳ*_*𝒩*_ on the back-transformed data mimic the features of the “true” heavy-tailed model *ℳ*_*G*_ very closely.

Since Normality is the single most typical and often required assumption in many areas of statistics, machine learning, and signal processing, future research can take many directions. From a theoretical perspective properties of Lambert W ×  *F*_*X*_ distributions viewed as a generalization of already well-known distributions *F*_*X*_ can be studied. This area will profit from existing literature on the Lambert W function, which has been discovered only recently by the statistics community. Empirical work can focus on transforming the data and comparing approximate Gaussian with joint heavy tail analyses. The comparisons in this work showed that approximate inference for Gaussianized data is comparable with the direct heavy tail modeling and so provides a simple tool to improve inference for heavy-tailed data in statistical practice.

I also provide the R package  Lambert W, publicly available at  CRAN, to facilitate the use of heavy tail Lambert W ×  *F*_*X*_ distributions in practice.

## Supplementary Material

The supplementary material contains proofs of the main results, details on the simulation setup and algorithms, and also lists some results for the two tails case.

## Figures and Tables

**Table 1:** Finite sample properties of ${\hat{\delta }}_{\text{MLE}}$. For each *N*, *δ* was estimated *n* = 1,000 times from a random sample **z**~Tukey's *h*. The left column for each *δ* shows bias, ${\bar{\hat{\delta }}}_{\text{MLE}}-\delta$; each right column shows the root mean square error (RMSE) times $\sqrt{N}$.

*N*	*δ* = 0		*δ* = 1/10		*δ* = 1/3		*δ* = 1/2	
10	0.025	0.191	−0.017	0.394	−0.042	0.915	−0.082	1.167
50	0.013	0.187	−0.010	0.492	−0.018	0.931	−0.016	1.156
100	0.010	0.200	−0.010	0.513	−0.009	0.914	−0.006	1.225
400	0.005	0.186	−0.003	0.528	0.000	0.927	−0.004	1.211
1000	0.003	0.197	0.000	0.532	−0.001	0.928	−0.001	1.203
2000	0.003	0.217	−0.001	0.523	0.000	0.935	−0.001	1.130

*N*	*δ* = 1		*δ* = 2		*δ* = 5			

10	−0.054	1.987	−0.104	3.384	−0.050	7.601		
50	−0.017	1.948	−0.009	3.529	0.014	7.942		
100	−0.014	2.024	−0.001	3.294	0.011	7.798		
400	0.001	1.919	−0.002	3.433	0.001	7.855		
1000	0.001	1.955	0.001	3.553	−0.001	7.409		
2000	0.001	1.896	0.000	3.508	−0.001	7.578		

**Table:** (a) Truly Gaussian data: *δ* = 0

*δ* = 0	Median	Gaussian MLE		IGMM				Lambert W MLE				NA
*N*		*μ*_*Y*_	*σ*_*Y*_	*μ*_*X*_	*σ*_*X*_	*δ*	*σ*_*Y*_	*μ*_*X*_	*σ*_*X*_	*δ*	*σ*_*Y*_	Ratio
50	0.00	0.00	0.98	0.00	0.97	0.02	0.99	0.00	0.96	0.02	0.98	0
100	0.00	0.00	0.99	0.00	0.98	0.01	1.00	0.00	0.97	0.01	0.99	0
1000	0.00	0.00	1.00	0.00	0.99	0.00	1.00	0.00	0.99	0.00	1.00	0

50	0.50	0.50	0.57	0.51	0.60	0.66	0.54	0.51	0.65	0.66	0.56	0
100	0.50	0.51	0.56	0.51	0.62	0.62	0.53	0.52	0.65	0.62	0.56	0
1000	0.50	0.49	0.52	0.49	0.62	0.56	0.52	0.49	0.63	0.56	0.52	0

50	1.24	1.01	0.72	1.01	0.76	0.21	0.73	1.02	0.78	0.26	0.72	0
100	1.25	1.02	0.70	1.02	0.76	0.23	0.70	1.03	0.78	0.26	0.70	0
1000	1.26	0.98	0.73	0.98	0.79	0.22	0.73	0.98	0.79	0.22	0.73	0

**Table:** (b) No fourth moments: *δ* = 1/3

*δ* = 1/3	Median	Gaussian MLE		IGMM				Lambert W MLE				NA
*N*		*μ*_*Y*_	*σ*_*Y*_	*μ*_*X*_	*σ*_*X*_	*δ*	*σ*_*Y*_	*μ*_*X*_	*σ*_*X*_	*δ*	*σ*_*Y*_	Ratio
50	0.00	0.00	1.98	0.00	1.07	0.29	∞	0.00	1.01	0.33	∞	0
100	0.00	0.00	2.03	0.00	1.04	0.31	∞	0.00	1.00	0.33	∞	0
1000	0.00	0.00	2.18	0.00	1.00	0.33	2.34	0.00	1.00	0.33	2.34	0

50	0.50	0.51	0.78	0.50	0.38	0.63	0.60	0.50	0.52	0.54	0.54	0
100	0.50	0.51	0.78	0.51	0.42	0.61	0.60	0.50	0.51	0.54	0.54	0
1000	0.48	0.51	0.77	0.51	0.47	0.56	0.55	0.51	0.50	0.53	0.52	0

50	1.27	2.21	6.56	1.44	1.45	1.10	NA	1.23	1.35	1.14	NA	0
100	1.30	2.33	11.28	1.43	1.42	1.12	NA	1.19	1.34	1.09	NA	0
1000	1.23	2.25	16.76	1.39	1.45	1.20	15.97	1.17	1.33	1.08	12.30	0

**Table:** (c) Non-existing mean: *δ* = 1

*δ* = 1	Median	Gaussian MLE		IGMM				Lambert W MLE				NA
*N*		*μ*_*Y*_	*σ*_*Y*_	*μ*_*X*_	*σ*_*X*_	*δ*	*σ*_*Y*_	*μ*_*X*_	*σ*_*X*_	*δ*	*σ*_*Y*_	Ratio
50	0.00	−0.10	24.6	−0.01	1.18	0.90	∞	0.00	1.01	0.99	∞	0
100	0.00	0.74	72.4	0.00	1.09	0.95	∞	0.00	1.01	0.99	∞	0
1000	0.00	3.84	348.1	0.00	1.01	1.00	∞	0.00	1.00	1.00	∞	0

50	0.53	0.52	1.0	0.51	0.34	0.65	1	0.51	0.52	0.52	1	0
100	0.50	0.52	1.0	0.51	0.38	0.63	1	0.50	0.53	0.53	1	0
1000	0.49	0.52	1.0	0.51	0.48	0.53	1	0.49	0.51	0.51	1	0

50	1.27	65.85	424.3	2.10	2.50	2.32	NA	1.19	1.70	2.16	NA	0
100	1.30	410.75	4050.2	2.01	2.28	2.59	NA	1.17	1.74	2.25	NA	0
1000	1.26	3307.58	104052.7	1.93	2.21	2.81	NA	1.11	1.64	2.18	NA	0

**Table:** (d) Extreme heavy tails: *δ* = 1.5

*δ* = 1.5	Median	Gaussian MLE		IGMM				Lambert W MLE				NA
*N*		*μ*_*Y*_	*σ*_*Y*_	*μ*_*X*_	*σ*_*X*_	*δ*	*σ*_*Y*_	*μ*_*X*_	*σ*_*X*_	*δ*	*σ*_*Y*_	Ratio
50	−0.02	6.84	309	−0.02	1.23	1.37	∞	−0.01	1.00	1.49	∞	0.01
100	0.00	−51.16	3080	−0.01	1.12	1.44	∞	0.00	1.01	1.50	∞	0.00
1000	0.00	176.13	14251	0.00	1.01	1.49	∞	0.00	1.00	1.50	∞	0.00

50	0.53	0.48	1	0.51	0.34	0.64	1	0.53	0.53	0.54	1	0.01
100	0.51	0.53	1	0.54	0.37	0.61	1	0.52	0.51	0.51	1	0.00
1000	0.50	0.50	1	0.50	0.47	0.54	1	0.49	0.53	0.52	1	0.00

50	1.32	1347.71	9261	2.57	3.20	3.12	NA	1.15	1.86	2.76	NA	0.01
100	1.33	42156.28	418435	2.39	2.87	3.44	NA	1.12	1.78	2.84	NA	0.00
1000	1.26	124462.82	3903629	2.18	2.66	3.67	NA	1.11	1.80	2.85	NA	0.00

**Table 3:** Summary statistics for observed (heavy-tailed) **y** and back-transformed (Gaussianized) data $x_{{\hat{\tau }}_{\text{MLE}}}$. *∗∗* stands for <10^−16^and *∗* for <2.2 · 10^−16^.

	**y** ~ *𝒞*(0,1) (Section 6.1)			**y** = S&P 500 (Section 6.2)	
	**y**	$x_{\hat{\tau }}$	$x_{\hat{\lambda }}$	**y**	$x_{\hat{\tau }}$
Min	−161.59	−3.16	0	−7.11	−2.42
Max	952.95	3.81	33.18	4.99	2.23
Mean	2.30	0.03	14.98	0.05	0.05
Median	0.04	0.04	14.96	0.04	0.04
Standard Deviation	46.980	1.06	1.20	0.95	0.71
Skewness	17.43	0.12	3.90	−0.30	−0.04
Kurtosis	343.34	3.21	161.75	7.70	2.93

Shapiro-Wilk	∗	0.71	∗∗	∗	0.24
Anderson-Darling	∗∗	0.51	∗∗	∗	0.18

**Table:** (a) Double-tail Lambert W  × Gaussian = Tukey's *hh* (S&P 500)

	Est.	se	*t*	Pr (>|*t*|)
*μ*_*X*_	0.06	0.015	3.66	0.00
*σ*_*X*_	0.71	0.016	44.00	0.00
*δ*_*ℓ*_	0.19	0.021	8.99	0.00
*δ*_*r*_	0.16	0.019	8.24	0.00

**Table:** (b) Skew *t* (S&P 500)

	Est.	se	*t*	Pr (>|*t*|)
*c*	0.10	0.061	1.65	0.10
*s*	0.67	0.017	38.47	0.00
*α*	−0.08	0.101	−0.77	0.44
*ν*	3.73	0.297	12.57	0.00

**Table:** (c) Lambert W  × Gaussian = Tukey's *h* (S&P 500)

	Est.	se	*t*	Pr (>|*t*|)
*μ*_*X*_	0.06	0.015	3.65	0.000
*σ*_*X*_	0.71	0.016	43.95	0.000
*δ*	0.17	0.016	11.05	0.000

**Table:** (d) Student's *t* (S&P 500)

	Est.	se	*t*	Pr (>|*t*|)
*c*	0.06	0.015	3.65	0.00
*s*	0.67	0.017	39.51	0.00
*ν*	3.72	0.295	12.61	0.00

**Table:** (e) Gaussian (S&P 500)

	Est.	se	*t*	Pr (>|*t*|)
*μ*_*Y*_	0.05	0.018	2.55	0.01
*σ*_*Y*_	0.95	0.013	74.57	0.00

**Table:** (f) Gaussian ($x_{{\hat{\tau }}_{\text{MLE}}}$)

	Est.	se	*t*	Pr (>|*t*|)
$\mu_{x_{\hat{\tau }}}$	0.05	0.013	3.81	0.00
$\sigma_{x_{\hat{\tau }}}$	0.71	0.009	74.57	0.00
