# Supplementary material for: The Lambert Way to Gaussianize Heavy-Tailed Data with the Inverse of Tukey's h Transformation as a Special Case
Source: ScientificWorldJournal. 2015 Aug 25;2015:909231. doi: 10.1155/2015/909231 (PMC4562338; doi:10.1155/2015/909231)
Supplement: Supplementary file 1 — The supplementary material contains proofs of the main results, details on the simulation setup and algorithms, and also lists some results for the two tails case. [file 909231.f1.pdf]

Supplementary Material of
“The Lambert Way to Gaussianize heavy tailed data with the inverse of Tukey’s h transformation as a special case”

Georg M. Goerg

A Auxiliary Results and Properties

A.1 Inverse Transformation $W_\delta(z)$

The function $W_\delta(z)$ is the building block of Lambert W $\times$ F_X distributions. This section lists useful properties of $W_\delta(z)$ as a function of z as well as a function of δ.

Properties A.1. *For* $\delta = 0$,

$$W_\delta(z_i) \mid_{\delta=0} = z_i, \quad W'(\delta z_i^2) \mid_{\delta=0} = z_i^2, \quad \text{and } W\left(\delta z_i^2\right) \mid_{\delta=0} = 0. \tag{37}$$

By definition $\frac{W_\delta(z)}{z} = e^{-\frac{\delta}{2} W_\delta(z)^2}$ and therefore

$$\log \frac{W_\delta(z)}{z} = -\frac{\delta}{2} W_\delta(z)^2 = -\frac{W(\delta z^2)}{2}. \tag{38}$$

Lemma A.2 (Derivative of $W_\delta(z)$ with respect to z)**.** *It holds*

$$\frac{d}{dz} W_\delta(z) = -\frac{W_\delta(z)}{z\left(1 + \delta W_\delta(z)^2\right)} = e^{-\frac{1}{2} W(\delta z^2)} \frac{1}{1 + W(\delta z^2)} \tag{39}$$

Proof. One of the many interesting properties of the Lambert W function relates to its derivative which satisfies

$$W'(z) = \frac{W(z)}{z(1 + W(z))} = \frac{1}{e^{W(z)}(1 + W(z))}, \quad z \neq 0, -1/e, \tag{40}$$

with $W'(0) = 1$ and $\lim_{z \to -1/e} W'(z) = \infty$. Hence,

$$\frac{d}{dz} \frac{W\left(\delta z^2\right)}{\delta} = W'\left(\delta z^2\right) \cdot 2z = \frac{W\left(\delta z^2\right)}{\delta z^2 \left(1 + W\left(\delta z^2\right)\right)} \cdot 2z = \frac{2W\left(\delta z^2\right)}{\delta z \left(1 + W\left(\delta z^2\right)\right)} \tag{41}$$

Therefore,

$$\begin{aligned} \frac{d}{dz} W_\delta(z) &= \frac{1}{2} \left(\frac{1}{\delta} W\left(\delta z^2\right)\right)^{-1/2} \cdot \frac{d}{dz} \frac{W\left(\delta z^2\right)}{\delta} && (42) \\ &= \frac{1}{2} \left(\frac{1}{\delta} W\left(\delta z^2\right)\right)^{-1/2} \cdot \frac{2W\left(\delta z^2\right)}{\delta z \left(1 + W\left(\delta z^2\right)\right)} && (43) \\ &= \frac{1}{\delta^{1/2}} \left(W\left(\delta z^2\right)\right)^{-1/2} \cdot \frac{W\left(\delta z^2\right)}{z \left(1 + W\left(\delta z^2\right)\right)} && (44) \end{aligned}$$

As $W\left(\delta z^2\right) = \delta u^2$ the last line simplifies to

$$\frac{1}{\delta^{1/2}} \frac{1}{\delta^{1/2} u} \cdot \frac{\delta u^2}{z\left(1+\delta u^2\right)} = \frac{u}{z\left(1+\delta u^2\right)}. \tag{45}$$

Now use again $u = W_\delta(z)$. □

Lemma A.3 (Derivative of $W_\delta(z)^2$ with respect to δ)**.** *For all $z \in \mathbb{R}$*

$$\frac{\partial}{\partial \delta}\left[W_\delta(z)\right]^2 = -\frac{1}{1+W\left(\delta z^2\right)} W_\delta\left(z\right)^4 \leq 0. \tag{46}$$

Proof. By definition $\left[W_\delta(z)\right]^2 = \frac{W\left(\delta z^2\right)}{\delta}$. Thus

$$\begin{aligned}
\frac{\partial}{\partial \delta} \frac{W\left(\delta z^2\right)}{\delta} &= \frac{\delta \frac{\partial}{\partial \delta} W\left(\delta z^2\right) - W\left(\delta z^2\right) \cdot 1}{\delta^2} &(47)\\
&= \frac{\delta W'\left(\delta z^2\right) z^2 - W\left(\delta z^2\right)}{\delta^2} &(48)\\
&= \frac{\delta \frac{W\left(\delta z^2\right)}{\delta z^2\left(1+W\left(\delta z^2\right)\right)} z^2 - W\left(\delta z^2\right)}{\delta^2} &(49)\\
&= \frac{\frac{W\left(\delta z^2\right)}{1+W\left(\delta z^2\right)} - W\left(\delta z^2\right)}{\delta^2} &(50)\\
&= \frac{\frac{-W\left(\delta z^2\right)^2}{1+W\left(\delta z^2\right)}}{\delta^2} &(51)\\
&= -\frac{1}{1+W\left(\delta z^2\right)}\left[W_\delta(z)\right]^4. &(52)
\end{aligned}$$

Since both terms are non-negative for all $z \in \mathbb{R}$, the result follows. □

This means that $W_\delta(z)^2$ is a decreasing function in δ for every $z \in \mathbb{R}$, i.e., the more we remove heavy tails the more z gets shrinked (non-linearly) towards $0 = \lim_{\delta \to \infty} W_\delta(z)$. In particular, $\left[W_\delta(z)\right]^2 < z^2 \Leftrightarrow \frac{W_\delta(z)}{z} < 1$ and $\frac{W_{\delta+\varepsilon}(z)}{z} < \frac{W_\delta(z)}{z}$for $\delta \geq 0$ and $\varepsilon > 0$.

Lemma A.4 (Derivative of $W_\delta(z)$ with respect to δ)**.** *It holds*

$$\frac{\partial}{\partial \delta} W_\delta\left(z\right) = -\frac{1}{2} \frac{1}{1+W\left(\delta z^2\right)} W_\delta\left(z\right)^3 \tag{53}$$

Proof.

$$\begin{aligned}
\frac{\partial}{\partial \delta} W_\delta\left(z\right) &= \operatorname{sgn}(z) \frac{\partial}{\partial \delta}\left(\frac{W\left(\delta z^2\right)}{\delta}\right)^{1/2} &(54)\\
&= \operatorname{sgn}(z) \frac{1}{2}\left(\frac{W\left(\delta z^2\right)}{\delta}\right)^{-1/2} \frac{\partial}{\partial \delta} \frac{W\left(\delta z^2\right)}{\delta} &(55)\\
&= \frac{1}{2} \frac{1}{W_\delta(z)} \frac{\partial}{\partial \delta}\left[W_\delta(z)\right]^2 &(56)\\
&= -\frac{1}{2} \frac{1}{1+W\left(\delta z^2\right)} W_\delta\left(z\right)^3, &(57)
\end{aligned}$$

where the last line follows by Lemma A.3. □

A.2 Penalty $\log R(\delta; z_i)$ for Standard Gaussian Input

For $\mu_X = 0$ and $\sigma_X = 1$ the penalty equals ($y_i = z_i$)

$$R(\delta; z_i) = \frac{W_\delta(z_i)}{z_i\left[1 + \delta\left(W_\delta(z_i)\right)^2\right]} = \frac{W_\delta(z_i)}{z_i\left[1 + W(\delta z_i^2)\right]} \tag{58}$$

and thus

$$\log R(\delta; z_i) = \log\frac{W_\delta(z_i)}{z_i} - \log\left[1 + W\left(\delta z_i^2\right)\right] \tag{59}$$

$$= -\frac{W(\delta z_i^2)}{2} - \log\left[1 + W\left(\delta z_i^2\right)\right] \tag{60}$$

Lemma A.5 (Derivative of $\log R(\delta; z)$ with respect to δ)**.** *For all $\delta \geq 0$ and all $z \in \mathbb{R}$*

$$\frac{\partial \log R(\delta; z)}{\partial \delta} = -z^2 W'(\delta z^2)\left(\frac{1}{2} + \frac{1}{1 + W(\delta z^2)}\right) \leq 0. \tag{61}$$

Proof. We have

$$\frac{\partial \log R(\delta; z)}{\partial \delta} = \frac{1}{W_\delta(z)}\frac{\partial W_\delta(z)}{\partial \delta} - \frac{1}{1 + W(\delta z^2)}W'(\delta z^2)z^2 \tag{62}$$

$$\overset{\text{Lemma A.4}}{=} \frac{1}{W_\delta(z)}\left(-\frac{1}{2}\frac{1}{1 + W(\delta z^2)}W_\delta(z)^3\right) - \frac{1}{1 + W(\delta z^2)}W'(\delta z^2)z^2 \tag{63}$$

$$= -\frac{1}{1 + W(\delta z^2)}\left(\frac{1}{2}W_\delta(z)^2 + W'(\delta z^2)z^2\right) \tag{64}$$

Using $W'(\delta z^2) = \frac{W(\delta z^2)}{\delta z^2(1 + W(\delta z^2))}$ and re-factorizing gives (61).

□

A.3 Gaussian Log-Likelihood at $W_\delta(z)$

Lemma A.6. *For all $z \in \mathbb{R}$ and for $\delta \geq 0$*

$$\frac{\partial}{\partial \delta}\ell(\mu_X = 0, \sigma_X = 1; W_\delta(z)) = \frac{1}{2}\frac{1}{1 + W(\delta z^2)}\left[W_\delta(z)\right]^4 \geq 0. \tag{65}$$

Proof. The log of the standard Gaussian pdf evaluated at $W_\delta(z)$ simplifies to

$$\log\frac{1}{\sqrt{2\pi}}e^{-\frac{1}{2}[W_\delta(z)]^2} = \log\frac{1}{\sqrt{2\pi}} - \frac{1}{2}\left[W_\delta(z)\right]^2. \tag{66}$$

The rest follows by Lemma A.3. □

Lemma A.6 shows that increasing δ always increases the input log-likelihood $\ell(\delta; \mathbf{u}_\delta = W_\delta(\mathbf{z}))$ - see also Fig. 6b. For $\delta \to \infty$ the Gaussianized $\mathbf{u}_\delta$ goes to $\mathbf{0}$, which trivially maximizes a Gaussian likelihood with $\mu_X = 0$.

B Proofs

B.1 Inverse Transformation

Proof of Lemma 2.5. Without loss of generality assume that $\mu_X = 0$ and $\sigma_X = 1$. Squaring (2) and multiplying by δ yields

$$\delta Z^2 \quad = \quad \delta U^2 \exp\left(\delta U^2\right) \tag{67}$$

The inverse of (67) is by definition the Lambert W function [45]

$$W(z) \exp W(z) = z, \quad z \in \mathbb{C}. \tag{68}$$

$W(z)$ is bijective for $z \geq 0$. Since $\delta U^2 \geq 0$ for all $\delta \geq 0$, applying $W(\cdot)$ to (67), dividing by δ, and taking the square root gives

$$U \quad = \quad \pm\sqrt{\frac{W\left(\delta Z^2\right)}{\delta}} \tag{69}$$

Since $\exp\left(\frac{\delta}{2}U^2\right) > 0$ for all $\delta \in \mathbb{R}$ and all U, it follows that $Z = U \exp\left(\frac{\delta}{2}U^2\right)$ and U must have the same sign, which concludes the proof. □

B.2 Cdf and Pdf

Proof of Theorem 2.7. By definition,

$$\begin{aligned} G_Y(y) = \mathbb{P}(Y \leq y) = \mathbb{P}\left(\left\{U \exp\left(\frac{\delta}{2}U^2\right)\right\} \sigma_X + \mu_X \leq y\right) \\ = \mathbb{P}\left(U \exp\left(\frac{\delta}{2}U^2\right) \leq z\right) = \mathbb{P}\left(U \leq W_\delta(z)\right) \\ = F_U\left(U \leq W_\delta(z)\right). \end{aligned}$$

Taking the derivative with respect to y gives

$$\begin{aligned} \frac{d}{dy} G_Y(y \mid \boldsymbol{\beta}, \delta) = f_X(W_\delta(z)\sigma_X + \mu_X \mid \boldsymbol{\beta}) \cdot \sigma_X \frac{d}{dy} W_\delta\left(\frac{y - \mu_X}{\sigma_X}\right) \\ = f_U(W_\delta(z) \mid \boldsymbol{\beta}) \cdot \sigma_X \frac{1}{\sigma_X} \frac{d}{dz} W_\delta\left(\frac{y - \mu_X}{\sigma_X}\right) \\ = f_U(W_\delta(z) \mid \boldsymbol{\beta}) \cdot \frac{d}{dz} W_\delta(z). \end{aligned}$$

Using Lemma A.2 yields (14). □

B.3 MLE for δ

Lemma B.1 (Derivative of the Lambert W $\times$ Gaussian log-likelihood)**.** *We have*

$$D(\delta; \mathbf{z}) := \frac{\partial}{\partial \delta} \ell(\delta; \mathbf{z}) = \sum_{i=1}^{N} z_i^2 W'(\delta z_i^2) \left(\frac{1}{2} W_\delta\left(z_i\right)^2 - \left(\frac{1}{2} + \frac{1}{1 + W\left(\delta z_i^2\right)} \right) \right) \tag{70}$$

$$= \frac{1}{2} \sum_{i=1}^{N} \frac{W_\delta\left(z_i\right)^4}{1 + \delta W_\delta\left(z_i\right)^2} - \sum_{i=1}^{N} \frac{W_\delta\left(z_i\right)^2}{1 + \delta W_\delta\left(z_i\right)^2} \left(\frac{1}{2} + \frac{1}{1 + \delta W_\delta\left(z_i\right)^2} \right) \tag{71}$$

$$= \frac{1}{2} \sum_{i=1}^{N} \frac{W_\delta\left(z_i\right)^4}{1 + W(\delta z_i^2)} - \sum_{i=1}^{N} \frac{W_\delta\left(z_i\right)^2}{1 + W(\delta z_i^2)} \left(\frac{1}{2} + \frac{1}{1 + W\left(\delta z_i^2\right)} \right). \tag{72}$$

Proof. Apply Lemmas A.5 and A.6 to $\frac{\partial}{\partial \delta} \ell(\delta; \mathbf{z}) = \frac{\partial}{\partial \delta} \log R\left(\delta; z\right) + \frac{\partial}{\partial \delta} \ell(\mu_X = 0, \sigma_X = 1; W_\delta(z))$. □

Proof sketch of Theorem 4.1. a) If condition (34) holds, then $D(\delta; \mathbf{z}) < 0$ at $\delta = 0$ and stays negative for all $\delta > 0$. Hence the maximizer occurs at the boundary $\delta = 0$.

b) If (34) does not hold, then $D(\delta = 0; \mathbf{z}) > 0$, decreases in δ and crosses the zero line (one candidate for $\widehat{\delta}_{MLE}$ occurs here).

c) As δ gets larger, $D(\delta; \mathbf{z})$ reaches a minimum (negative value) and starts increasing. However, for $\delta \to \infty$ the derivative approaches zero from below and never equals zero again; thus $\widehat{\delta}_{MLE}$ is unique.

□

Proof of Theorem 4.1. a) The log-likelihood is increasing at $\delta = 0$ if and only if (set $\delta = 0$ in (72) and use Property A.1)

$$\sum_{i=1}^{N} z_i^4 > 3 \sum_{i=1}^{N} z_i^2. \tag{73}$$

Eq. (73) means that transforming the data (choosing $\widehat{\delta} > 0$) increases the overall likelihood only if the data is heavy-tailed enough. As the sum of squares is not squared again condition (73) is not equivalent for the data having empirical kurtosis larger than 3.

b) If (73) does not hold, then $\widehat{\delta}_{MLE}$ must satisfy $D(\delta; \mathbf{z}) \mid_{\delta = \widehat{\delta}_{MLE}} = 0$ from (70) in Lemma B.1. It remains to be shown that this equation has (at least) one positive solution.

i) Since $\lim_{\delta \to \infty} W_\delta(z) = 0$ for all $z \in \mathbb{R}$, (72) is also true in the limit; however, we can ignore this solution as we require $\widehat{\delta}_{MLE} \in \mathbb{R}$.

ii) By continuity and $\lim_{\delta \to \infty} W_\delta(z) = 0$, for sufficiently large δ_M, $W_{\delta_M}(z_i) < 1$ for all $z_i \in \mathbb{R}$. Hence $W_{\delta_M}(z_i)^4 < W_{\delta_M}(z_i)^2$ and therefore

$$\frac{1}{2} \sum_{i=1}^{N} \frac{W_\delta\left(z_i\right)^4}{1 + \delta W_\delta\left(z_i\right)^2} < \frac{1}{2} \sum_{i=1}^{N} \frac{W_\delta\left(z_i\right)^2}{1 + \delta W_\delta\left(z_i\right)^2} \tag{74}$$

$$< \sum_{i=1}^{N} \frac{W_\delta\left(z_i\right)^2}{1 + \delta W_\delta\left(z_i\right)^2} \left(\frac{1}{2} + \frac{1}{1 + \delta W_\delta\left(z_i\right)^2} \right) \quad \text{for } \delta \geq \delta_M, \tag{75}$$

showing that $D(\delta; \mathbf{z}) \mid_{\delta \geq \delta_M} < 0$. That is, $D(\delta; \mathbf{z})$ approaches 0 from below for $\delta \to \infty$.

iii) By continuity and $D(\delta; \mathbf{z}) \mid_{\delta=0} > 0$ (if (73) does not hold), it must cross the $D(\delta; \mathbf{z}) = 0$ line at least once in the interval $(0, \delta_M)$, proving the existence of $\widehat{\delta}_{MLE}$.

c) The log-likelihood can be decomposed in

$$\ell(\delta; \mathbf{z}) \propto \underbrace{-\frac{1}{2}\sum_{i=1}^{N}\left[W_\delta(z_i)\right]^2}_{\ell(\mu_X=0, \sigma_X=1; W_\delta(\mathbf{z}))} + \underbrace{\sum_{i=1}^{N} \log \frac{W_\delta(z_i)}{z_i} - \log\left[1 + W\left(\delta z_i^2\right)\right]}_{\mathcal{R}(\delta; \mathbf{z})}. \tag{76}$$

Lemmas A.5 and A.6 show that $\mathcal{R}(\delta; \mathbf{z})$ is monotonically decreasing and $\ell(\mu_X = 0, \sigma_X = 1; W_\delta(\mathbf{z}))$ is monotonically increasing in δ.

Furthermore, $\lim_{\delta \to \infty} \ell(\mu_X = 0, \sigma_X = 1; W_\delta(\mathbf{z})) = 0$, that is the input likelihood is monotonically increasing but bounded from above (by $0 = \log 1$). On the other hand the penalty is decreasing without bounds

$$\lim_{\delta \to \infty} \mathcal{R}(\delta; \mathbf{z}) = \sum_{i=1}^{N} \lim_{\delta \to \infty} \log \frac{W_\delta(z_i)}{z_i} - \sum_{i=1}^{N} \lim_{\delta \to \infty} \log\left[1 + W\left(\delta z_i^2\right)\right] = -\infty \tag{77}$$

Thus their sum attains a global maximum either at the unique mode of $\ell(\delta; \mathbf{z})$ or at the boundary $\delta = 0$ - see also Fig. 6b.

□

C Details on IGMM

Here I present an iterative method to obtain $\widehat{\tau}$, which builds on the input/output aspect and theoretical properties of the input X. For example, if a random variable should be exponentially distributed but the observed data shows heavier tails, then it is natural to estimate $\sigma_X = \lambda^{-1}$ and δ such that the back-transformed data has skewness 2, as this is a general property of exponential random variables – independent of the rate parameter λ; to remove heavy tails from an otherwise symmetric $\mathbf{y}$ a natural choice for τ is such that the back-transformed data $\mathbf{x}_\tau$ has sample kurtosis 3; or for uniform input, τ should be such that $\mathbf{x}_\tau$ has a flat density estimate.

Here I describe the estimator for τ to remove heavy-tails in location-scale data, in the sense that the kurtosis of the input should equal 3. It can be easily adapted to match other properties of the input as outlined above.

For a moment assume that $\mu_X = \mu_X^{(0)}$ and $\sigma_X = \sigma_X^{(0)}$ are known and fixed; only δ has to be estimated. A natural choice for δ is the one that results in back transformed data $\mathbf{x}_\tau$ ($\tau = (\mu_X^{(0)}, \sigma_X^{(0)}, \delta)$) with sample kurtosis $\widehat{\gamma}_2(\mathbf{x}_\tau)$ equal to the theoretical kurtosis $\gamma_2(X)$. Formally,

$$\widehat{\delta}_{\text{GMM}} = \arg\min_{\delta} ||\gamma_2(X) - \widehat{\gamma}_2(\mathbf{x}_\tau)||, \tag{78}$$

where $||\cdot||$ is a proper norm in $\mathbb{R}$.

While the concept of this estimator is identical to its skewed version [21], it has one important advantage: the inverse transformation is bijective. Thus here we do not have to consider "lost" data points from the non-principal branch of the Lambert W function when applying the inverse transformation.

Algorithm 1 Find optimal δ : `delta_GMM()` in the `LambertW` package.

Input: data vector $\mathbf{z}$; theoretical kurtosis $\gamma_2(X)$
Output: $\widehat{\delta}_{GMM}$ as in (78)

$\widehat{\delta}_{GMM} = \arg\min_{\delta} ||\widehat{\gamma}_2(\mathbf{u}_\delta) - \gamma_2(X)||$, where $\mathbf{u}_\delta = W_\delta(\mathbf{z})$ subject to $\delta \geq 0$
return $\widehat{\delta}_{GMM}$

Algorithm 2 Iterative Generalized Method of Moments (IGMM) : `IGMM()` in the `LambertW` package.

Input: data vector $\mathbf{y}$; tolerance level *tol*; theoretical kurtosis $\gamma_2(X)$
Output: IGMM parameter estimate $\widehat{\tau}_{\mathrm{IGMM}} = (\widehat{\mu}_X, \widehat{\sigma}_X, \widehat{\delta})$

Starting values: $\tau^{(0)} = (\mu_X^{(0)}, \sigma_X^{(0)}, \delta^{(0)})$, where $\mu_X^{(0)} = \tilde{\mathbf{y}}$ and $\sigma_X^{(0)} = \overline{\sigma}_y \cdot \left(\frac{1}{\sqrt{(1-2\delta^{(0)})^{3/2}}}\right)^{-1}$ are the sample median and standard deviation of $\mathbf{y}$ divided by the standard deviation factor (see also (18)), respectively. $\delta^{(0)} = \frac{1}{66}\left(\sqrt{66\widehat{\gamma}_2(\mathbf{y}) - 162} - 6\right) \rightarrow$ see (21) for details.
$k = 0$
Set $\tau^{(-1)} = \tau^0 + 2 \cdot tol$ to start the while loop.
while $\left|\left|\tau^{(k)} - \tau^{(k-1)}\right|\right| > tol$ **do**
  $\mathbf{z}^{(k)} = (\mathbf{y} - \mu_X^{(k)})/\sigma_X^{(k)}$
  Pass $\mathbf{z}^{(k)}$ to Algorithm 1 $\longrightarrow \delta^{(k+1)}$
  Back-transform $\mathbf{z}^{(k)}$ to $\mathbf{u}^{(k+1)} = W_{\delta^{(k+1)}}(\mathbf{z}^{(k)})$; compute $\mathbf{x}^{(k+1)} = \mathbf{u}^{(k+1)}\,\sigma_X^{(k)} + \mu_X^{(k)}$
  Update parameters: $\mu_X^{(k+1)} = \overline{\mathbf{x}}_{k+1}$ and $\sigma_X^{(k+1)} = \widehat{\sigma}_{x_{k+1}}$
  $\tau^{(k+1)} = (\mu_X^{(k+1)}, \sigma_X^{(k+1)}, \delta^{(k+1)})$
  $k = k + 1$
return $\tau_{IGMM} = \tau^{(k)}$

Discussion of Algorithm 1: The kurtosis of Y as a function of δ is continuous and monotonically increasing (see (19)). Also $u = W_\delta(z)$ has a smaller slope than the identity $u = z$, and the slope is decreasing as δ is increasing. Thus if the kurtosis of the original data is larger than the target kurtosis of the back-transformed data, $\widehat{\gamma}_2(\mathbf{y}) > \gamma_2(X)$, then there always exists a $\delta^{(*)}$ that achieves $\widehat{\gamma}_2(\mathbf{x}_{\tau^*}) \equiv \gamma_2(X)$. By the re-parametrization $\tilde{\delta} = \log \delta$ the bounded optimization problem can be solved by standard (unconstrained) optimization algorithms.

In practice, μ_X and σ_X are rarely known but also have to be estimated. As $\mathbf{y}$ is shifted and scaled *ahead of* the back-transformation $W_\delta(\cdot)$, the initial choice of μ_X and σ_X affects the optimal choice of δ. Therefore the optimal triple $\widehat{\tau} = (\widehat{\mu}_X, \widehat{\sigma}_X, \widehat{\delta})$ must be obtained iteratively.

Discussion of Algorithm 2: Algorithm 2 first computes $\mathbf{z}^{(k)} = (\mathbf{y} - \mu_X^{(k)})/\sigma_X^{(k)}$ using $\mu_X^{(k)}$ and $\sigma_X^{(k)}$ from the previous step. This normalized output can then be passed to Algorithm 1 to obtain an updated $\delta^{(k+1)} = \widehat{\delta}_{GMM}$. Using this new $\delta^{(k+1)}$ one can back-transform $\mathbf{z}^{(k)}$ to $\mathbf{u}^{(k+1)} = W_{\delta^{(k+1)}}(\mathbf{z}^{(k)})$, and consequently obtain a better approximation to the "true" latent $\mathbf{x}$ by $\mathbf{x}^{(k+1)} = \mathbf{u}^{(k+1)}\,\sigma_X^{(k)} + \mu_X^{(k)}$. However, $\delta^{(k+1)}$ - and therefore $\mathbf{x}^{(k+1)}$ - has been obtained using $\mu_X^{(k)}$ and $\sigma_X^{(k)}$, which are not necessarily the most accurate estimates in light of the updated approximation $\widehat{\mathbf{x}}_{(\mu_X^{(k)}, \sigma_X^{(k)}, \delta^{(k+1)})}$. Thus Algorithm 2 computes new estimates $\mu_X^{(k+1)}$ and $\sigma_X^{(k+1)}$ by the sample mean and standard deviation of $\widehat{\mathbf{x}}_{(\mu_X^{(k)}, \sigma_X^{(k)}, \delta^{(k+1)})}$, and starts another iteration by passing the updated normalized output

Algorithm 3 Random sample generation : `rLambertW()` in the `LambertW` package.

Input: number of samples n; parameter vector θ; input distribution $F_X(x)$ with finite mean and variance

Output: random sample $(y_1, \ldots, y_n)$ of a Lambert W $\times$ F_X random variable.

1: Draw n random samples $\mathbf{x} = (x_1, \ldots, x_n) \sim F_X(x)$.
2: Compute $\mu_X = \mu_X(\boldsymbol{\beta})$ and $\sigma_X = \sigma_X(\boldsymbol{\beta})$ (for scale family set $\mu_X = 0$, for non-central, non-scaled also set $\sigma_X = 1$)
3: Compute normalized $\mathbf{u} = (\mathbf{x} - \mu_X)/\sigma_X$.
4: $\mathbf{z} = \mathbf{u} \exp\left(\frac{\delta}{2}\mathbf{u}^2\right)$
5: **return** $\mathbf{y} = \mathbf{z}\sigma_X + \mu_X$

$\mathbf{z}^{(k+1)} = \frac{\mathbf{y}-\mu_X^{(k+1)}}{\sigma_X^{(k+1)}}$ to Algorithm 1 to obtain a new $\delta^{(k+2)}$. It returns the optimal $\widehat{\tau}_{\text{IGMM}}$ once convergence has been reached, i.e., if $\left|\left|\tau^{(k)} - \tau^{(k+1)}\right|\right| < tol$.

D The Asymmetric Double-tail Case

Corollary D.1 (Inverse transformation for Tukey's hh)**.** *The inverse of* (3) *is*

$$W_{\delta_\ell,\delta_r}(z) = \begin{cases} W_{\delta_\ell}(z), & \text{if } z \leq 0, \\ W_{\delta_r}(z), & \text{if } z > 0. \end{cases} \quad (79)$$

Figure 3b shows $W_{\delta_\ell,\delta_r}(z)$ for $\delta_l = 0$ and $\delta_r = 1/10$. The transformation in Fig. 3a generates a right heavy tail version of U (x-axis) by stretching only the positive axis (y-axis). By definition $W_{\delta_\ell,\delta_r}(z)$ removes the heavier right tail in Z (positive y-axis). Figure 3c shows how $W_\delta(z)$ operates for various degrees of heavy tails and $z \in [0, 3]$. If δ is close to zero, then also $W_\delta(z) \approx z$; for larger δ, the inverse maps z to (much) smaller u.

Corollary D.2. *The cdf and pdf of Z in* (3) *equal*

$$G_Z(z \mid \boldsymbol{\beta}, \delta_\ell, \delta_r) = \begin{cases} G_Z(z \mid \boldsymbol{\beta}, \delta_\ell), & \text{if } z \leq 0, \\ G_Z(z \mid \boldsymbol{\beta}, \delta_r), & \text{if } z > 0, \end{cases} \quad (80)$$

and

$$g_Z(z \mid \boldsymbol{\beta}, \delta) = \begin{cases} g_Z(z \mid \boldsymbol{\beta}, \delta_\ell), & \text{if } z \leq 0, \\ g_Z(z \mid \boldsymbol{\beta}, \delta_r), & \text{if } z > 0. \end{cases} \quad (81)$$

Remark D.3 (IGMM for double-tail Lambert W $\times$ F_X)**.** *For a double-tail fit the one-dimensional optimization in Algorithm 1 has to be replaced with a two-dimensional optimization*

$$\left(\widehat{\delta}_\ell, \widehat{\delta}_r\right)_{\text{GMM}} = \arg\min_{\delta_\ell,\delta_r} \left|\left|\gamma_2(X) - \widehat{\gamma}_2(\mathbf{x}_{(\mu_X^*,\sigma_X^*,\delta_\ell,\delta_r)})\right|\right|. \quad (82)$$

Algorithm 2 remains unchanged, except for replacing $W_{\delta^{(k+1)}}(\mathbf{z}^{(k)})$ with $W_{\delta_\ell^{(k+1)},\delta_r^{(k+1)}}(\mathbf{z}^{(k)})$ and $\tau = (\mu_X, \sigma_X, \delta_\ell, \delta_r)$.
